# Comparative Transcriptomics Profiling of Perennial Ryegrass Infected with Wild Type or a Δ*velA Epichloë festucae* Mutant Reveals Host Processes Underlying Mutualistic versus Antagonistic Interactions

**DOI:** 10.3390/jof9020190

**Published:** 2023-02-01

**Authors:** Mostafa Rahnama, Paul Maclean, Damien J. Fleetwood, Richard D. Johnson

**Affiliations:** 1 Department of Biology, Tennessee Tech University, Cookeville, TN 38505, USA; 2 AgResearch, Grasslands Research Centre, Palmerston North 4442, New Zealand; 3 Biotelliga Ltd., Auckland 1151, New Zealand

**Keywords:** plant–microbe interactions, endophytes, comparative transcriptomics, velvet genes

## Abstract

*Epichloë* species form bioprotective endophytic symbioses with many cool-season grasses, including agriculturally important forage grasses. Despite its importance, relatively little is known about the molecular details of the interaction and the regulatory genes involved. VelA is a key global regulator in fungal secondary metabolism and development. In previous studies, we showed the requirement of *velA* for *E. festucae* to form a mutualistic interaction with *Lolium perenne*. We showed that VelA regulates the expression of genes encoding proteins involved in membrane transport, fungal cell wall biosynthesis, host cell wall degradation, and secondary metabolism, along with several small-secreted proteins in *Epichloë festucae*. Here, by a comparative transcriptomics analysis on perennial ryegrass seedlings and mature plants, which are endophyte free or infected with wild type (mutualistic interaction) or mutant Δ*velA E. festucae* (antagonistic or incompatible interaction), regulatory effects of the endophytic interaction on perennial ryegrass development was studied. We show that Δ*velA* mutant associations influence the expression of genes involved in primary metabolism, secondary metabolism, and response to biotic and abiotic stresses compared with wild type associations, providing an insight into processes defining mutualistic versus antagonistic interactions.

## 1. Introduction

Fungi of the genus *Epichloë* form endophytic symbioses with cool-season grasses of the sub-family Pooideae, including agriculturally important forages such as tall fescue (*Festuca arundinacea*) and perennial ryegrass (*Lolium perenne*) and are widely distributed in natural grasslands [1,2,3]. During this interaction, fungi receive all their nutrients from the host plant and use the host seed as a means of dissemination, while protecting the plant from a range of biotic and abiotic stresses. Resistance to herbivory from insects is the best characterised of these and is mediated by production of four different classes of alkaloids: indole-diterpenes, ergot alkaloids, lolines, and peramine [4,5]. Recently, the *Epichloë festucae*–perennial ryegrass (PRG) interaction has been used as a model system to understand mutualistic versus pathogenic (antagonistic) interactions using different *E. festucae* mutants [6,7,8]. One such study using a strain mutated in the *velA* gene (velvet) showed that velvet is required for fungal biology and development and for the establishment and maintenance of the mutualistic interaction of the fungus with its host PRG during both the early (seedling) and late (mature) stages of the interaction [6]. In addition, in a comparative transcriptomics study, we identified a set of genes regulated by VelA that underlay the mutualistic interaction in *E. festucae* [9].

Although most transcriptomics studies involving *Epichloë*–grass interactions have focused on fungal genes, there are some studies that have examined host gene expression during the interaction. These studies mostly focused on comparing endophyte-free grasses with infected grasses [10,11,12,13]. In these studies, infected plants showed up-regulation of genes associated with cellular protein transport, protein synthesis, and turnover, and down-regulation of genes associated with carbohydrate metabolism [11,12]. In another study, transcriptomics of *E. festucae*–PRG using different host tissues and developmental stages were compared [13]. Their results showed moderate increases in the expression of PRG genes involved in hormone biosynthesis and perception, as well as stress and pathogen resistance, but down-regulation of genes involved in photosynthesis [13]. Down-regulation of genes involved in photosynthesis was also shown by Johnson et al. (2003) and Khan et al. (2010) for tall fescue (*Lolium arundinaceum*) and PRG associations, respectively [10,11]. Symbiotic interaction of tall fescue with *E. coenophiala* showed differential expression of genes mostly belonging to defence responses and abiotic stresses [14]. The same group showed that water deficit affected 38% of the plant transcripts and that endophyte infection conferred protection through influencing plant gene expression [15]. 

Based on our knowledge of the regulatory roles of VelA on the PRG-*Epichloë* symbiosis [6,9] we used mRNA-sequencing to compare the expression profiles of PRG, at two different development stages (seedlings and mature plants), infected with either wild type (compatible) or Δ*velA* mutant (incompatible) strains or endophyte free to identify host processes that may underlie these different compatibility outcomes. 

## 2. Materials and Methods

### 2.1. Sample Preparation 

For mature plant treatments, total RNA was extracted from three-months-old endophyte free (E-) and infected perennial ryegrass, *L. perenne* ‘Nui’, with wild type and Δ*velA E. festucae*, which had been previously generated in an earlier study [6]. The top 4 cm of the newest mature blade of plants from each treatment group were harvested into liquid nitrogen, with three replicates for each treatment. 

For the seedling treatments, endophyte-free seedlings (7–10 d old) of the *L. perenne* ‘Nui’ were inoculated with wild type and Δ*velA* mutant strains of *E. festucae.* After two weeks on PDA medium, inoculated seedlings were grown for a further two weeks under 16 h of 650 W/m^2^ light and 8 h of darkness and, after freezing in liquid nitrogen, samples from 4 cm upwards and 0.5 cm downwards from the meristem were harvested. We pooled 100 seedlings for each sample in three replicates for each treatment and RNA was extracted from each pool of seedlings. Besides these two E+ treatments, E- seedlings were also prepared in triplicate and pooled as described above. 

After determining RNA quality and quantity [9,16], sequencing was performed on an Illumina HiSeq4000 sequencer (paired end, 100-bp reads), as described by Rahnama et al [6,16]. 

### 2.2. HiSeq Results Analysis 

Gene sets of ryegrass (https://ryegrassgenome.ghpc.au.dk/DOWNLOAD/lope_V1.0/lope_V1.0_transcr_DNA.fasta, accessed on 1 April 2019) were mapped against the genome scaffold for ryegrass (downloaded from https://ryegrassgenome.ghpc.au.dk/DOWNLOAD/lope_V1.0/lope_V1.0.fasta, accessed on 1 April 2019) with Exonerate version 2.2.0 using the –est2genome model and keeping alignments scoring at least 50 percentage of the maximal score for each query. The target GFF option was used for the exon coordinates to be imported into RNA-star to enumerate the genes [17].

Reads were trimmed using flexbar version 2.4 [18] and mapped against the prepared database using RNA-star version 2.5.0c [17]. Non-directional counts of uniquely mapped read pairs were summed for each gene and analysed using the EdgeR package version 3.10.5 [19] in the R statistical software environment version 3.2.1. Quasi-likelihood negative binomial generalized linear models were generated from the counts within sample type. Fold changes and p-values were generated using Exact Tests for differences between two groups of Negative-Binomial Counts.

### 2.3. Functional Annotation

Perennial ryegrass transcript sequences were downloaded from https://ryegrassgenome.ghpc.au.dk/DOWNLOAD/lope_V1.0/lope_V1.0_transcr_DNA.fasta, accessed on 1 April 2019 and the Mercator tool (http://mapman.gabipd.org/web/guest/app/mercator, accessed on 1 April 2019) was used to bin all transcripts based on hierarchical ontologies after searching a variety of databases. Then, a MapMan mapping file was generated especially for perennial ryegrass. For pathway analysis, the MapMan tool was used based on the available protocol [20,21]

In addition, protein sequences for the perennial ryegrass (downloaded from https://ryegrassgenome.ghpc.au.dk/DOWNLOAD/lope_V1.0/lope_V1.0_transcr_PROT.fasta, accessed on 1 April 2019) were searched for matches against InterPro protein signature databases using InterProScan 5RC4, Swiss-Prot database, UniProt, and NCBI using BLASTP version 2.2.28+ and Blast2GO based on the settings of Rahnama et al [8,16]. 

### 2.4. General Bioinformatics Analyses

Venn diagrams were drawn using BioVenn online software [22]. Volcano plots were drawn using Tmisc package version 0.1.5 and devtools package version 1.11.1 [23] in R statistical software environment version 3.2.1 [24]. 

## 3. Results

### 3.1. General Description of RNA-Sequencing Results

**Figure 1:**
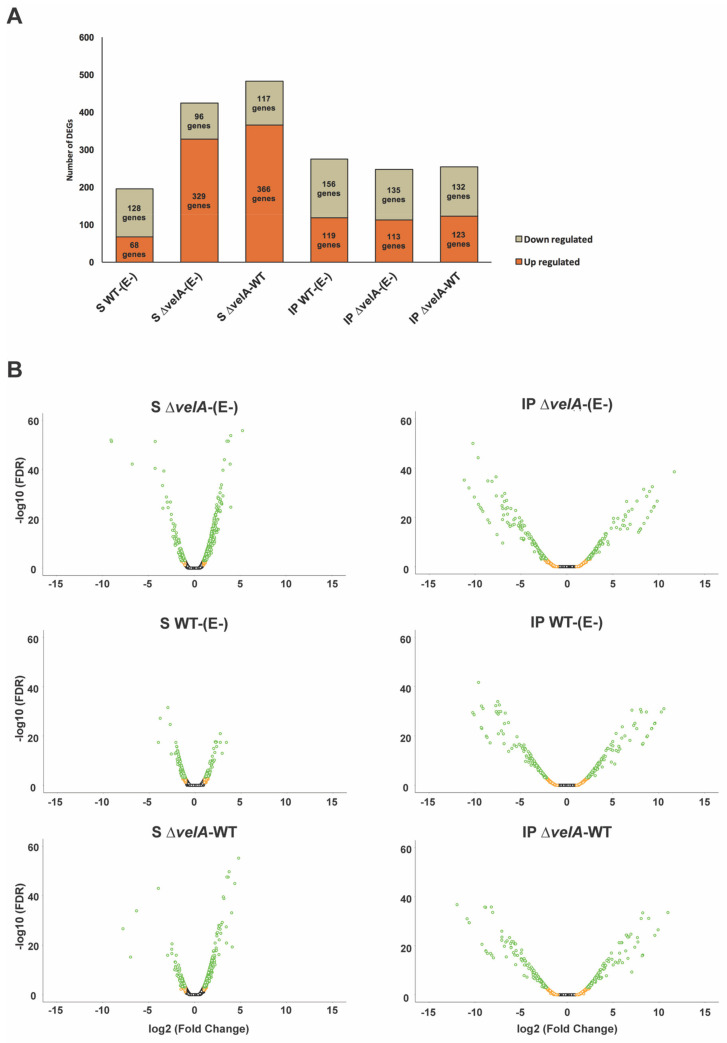

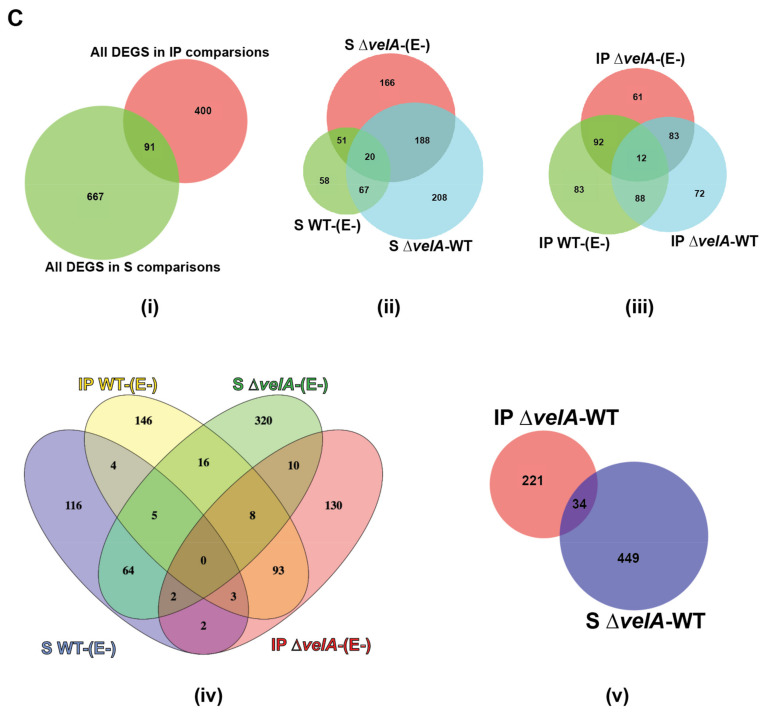
Distribution of deferentially expressed genes (DEGs) of perennial ryegrass (seedlings and mature plants) in response to wild type and Δ*velA* mutant *Epichloë festucae* infection. (**A**) The bar chart shows the number of DEGs up− or down−regulated in different comparisons. (**B**) The volcano plots of DEGs distribution by log_2_ fold change (logFC) and −log10 of FDR in three different comparisons. Black dots: FDR > 0.05, Red dots: FDR <= 0.05, orange dots: logFC >= 1, green dots: FDR <= 0.05 & logFC >= 1. (**C**) Venn diagram of common DEGs in different comparisons of (i) All DEGs in IP comparisons vs. All DEGs in S comparisons; (ii) S Δ*velA*-(E-) vs. S WT-(E-) vs. S Δ*velA*-WT; (iii) IP Δ*velA*-(E-) vs. IP WT-(E-) vs. IP Δ*velA*-WT; (iv) IP WT-(E-) vs. IP Δ*velA*-(E-) vs. S Δ*velA*-(E-) vs. S WT-(E-); (v) IP Δ*velA*-WT vs. S Δ*velA*-WT.

In total, 715,183,580 grass reads mapped to the ryegrass genome (Table S1). Genes with two times or greater fold differential expression and an FDR less than or equal to 0.05 were considered as differentially expressed genes (DEGs) in this study. In total, six comparisons were studied; three in PRG seedlings, including inoculated seedlings with wild type *E. festucae* versus endophyte-free seedlings (S WT-(E-)), inoculated seedlings with Δ*velA E. festucae* versus endophyte-free seedlings (S Δ*velA*-(E-)), and inoculated seedlings with Δ*velA* versus wild type *E. festucae* (S Δ*velA*-WT), and three in mature PRG plants, including infected plants with wild type *E. festucae* versus endophyte-free plants (IP WT-(E-)), infected plants with Δ*velA E. festucae* versus endophyte-free plants (IP Δ*velA*-(E-)), and infected plants with Δ*velA* versus wild type *E. festucae* (IP Δ*velA*-WT). In seedling comparisons, 1.09% (196 genes), 2.37% (425 genes), and 2.69% (483 genes) were differentially expressed in S WT-(E-), S Δ*velA*-(E-), and S Δ*velA*-WT comparisons, respectively (Figure 1A), with different ranges of fold changes (Figure 1B). In mature plant comparisons, 1.53% (275 genes), 1.38% (248 genes), and 1.42% (255 genes) were differentially expressed in IP WT-(E-), IP Δ*velA*-(E-), and IP Δ*velA*-WT comparisons, respectively (Figure 1A), with similar ranges of fold changes (Figure 1B). Interestingly, infecting seedlings with mutant fungi (S Δ*velA*-(E-)) had 2x more differentially expressed genes (425) compared with associations with the wild type (S WT-(E-) (196). For the mature associations, IP Δ*velA*-(E-) had 248 DE genes compared with IP WT-(E-) with 278 DE genes, of which only 104 of them were common (Figure 1Ciii).

There are 491 DEGs in at least one of the mature plant comparisons and 758 DEGs in at least one of the seedling comparisons; however, interestingly, only 91 genes are common between these two groups (Figure 1Ci). Studying common genes between the seedling comparisons showed that most of the DEGs in wild-type infected seedlings were unique compared with mutant infected seedlings, with only a small number of DEGs being common between them (Figure 1Cii). This is similar to mature plant comparisons (Figure 1Ciii). Additionally, comparing DEGs in S Δ*velA*-WT with IP Δ*velA*-WT (Figure 1Cv) showed that only 34 genes are in common. 

### 3.2. Functional Annotations of Differentially Expressed Ryegrass Genes

**Figure 2:**
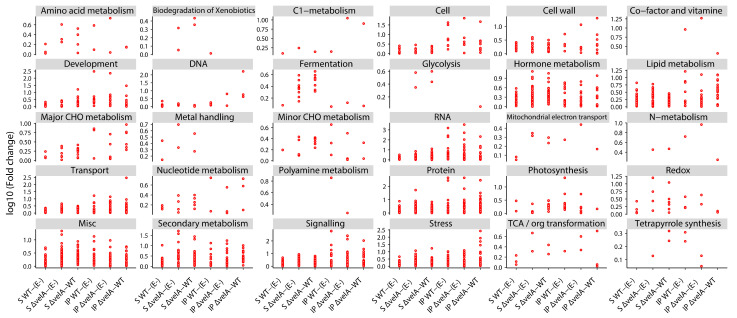
Fold change distribution of the genes in different metabolic pathways of ryegrass that at least have one gene that differentially expressed in one of the comparisons. Metabolic pathways categories resulted from MapMan.

The functions of DEGs were further analysed by categorising DEGs into manually curated bins using Mercator, followed by analysis of diagrammatic outputs generated by MapMan software. The results showed that inoculating ryegrass plants with wild type and Δ*velA E. festucae* mutants changed the expression of genes in 30 of 51 different metabolic pathways of ryegrass (Figure 2). The significant DEGs in different pathways associated with primary metabolism, secondary metabolism, and response to biotic and abiotic stresses were analysed in detail and are described below. 

### 3.3. Mutant Endophytes Change Primary Metabolism in Their Host Plants 

**Figure 3:**
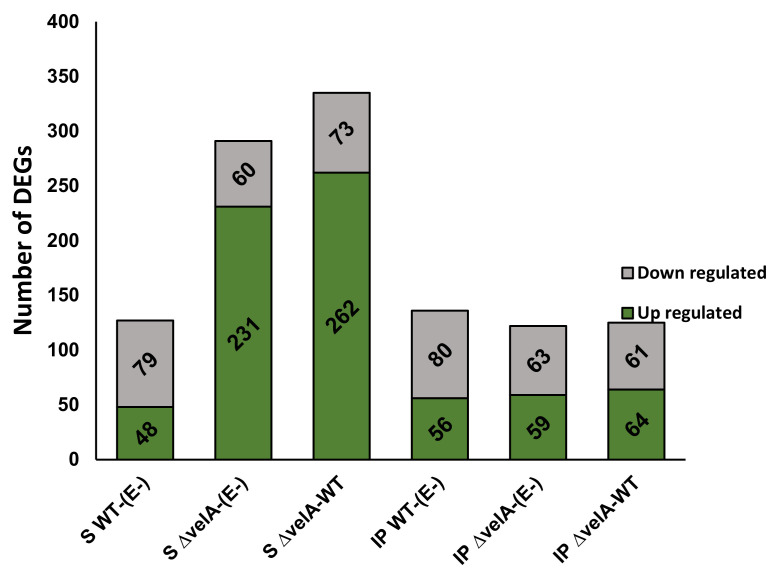
Number of DEGs that were categorised in primary metabolism resulting from MapMan analyses.

Most of the DEGs predicted to be involved in primary metabolism were up-regulated in Δ*velA* infected seedlings (231 of 291 and 262 of 335 genes in S Δ*velA*-(E-) and S Δ*velA*-WT comparisons, respectively), but for WT infected seedlings (S WT-(E-)), the opposite was seen, with 79 of 127 DEGs being down-regulated. Interestingly, in the mature associations, there was no particular direction of altered expression (Figure 3).

**Figure 4:**
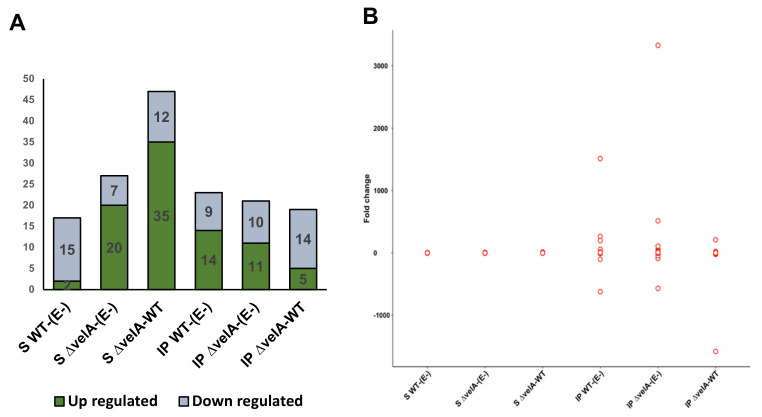
Distribution of predicted genes that encode enzymes involved in RNA metabolism (RNA transcription, regulation of transcription, RNA processing). (**A**) Percentage of DEGs per total predicted genes that encode enzymes involved in RNA metabolism (RNA transcription, regulation of transcription, RNA processing). (**B**) Fold change distribution of DEGs in RNA metabolism in different comparisons.

Of 650 genes predicted to encode enzymes involved in RNA metabolism (RNA transcription, regulation of transcription, RNA processing), 49 genes (7.5%) were differentially expressed at least in one of the seedling comparisons and 41 genes (6.3%) in one of the mature comparisons (Table S2). In the S Δ*velA*-WT comparison, most of the DEGs were up-regulated, but in IP Δ*velA*-WT comparisons, most of the genes were down-regulated (Figure 4A, Table S2). In mature comparisons, DEGs generally showed a much higher fold expression change compared with seedling comparisons (Figure 4B, Table S2).

**Figure 5:**
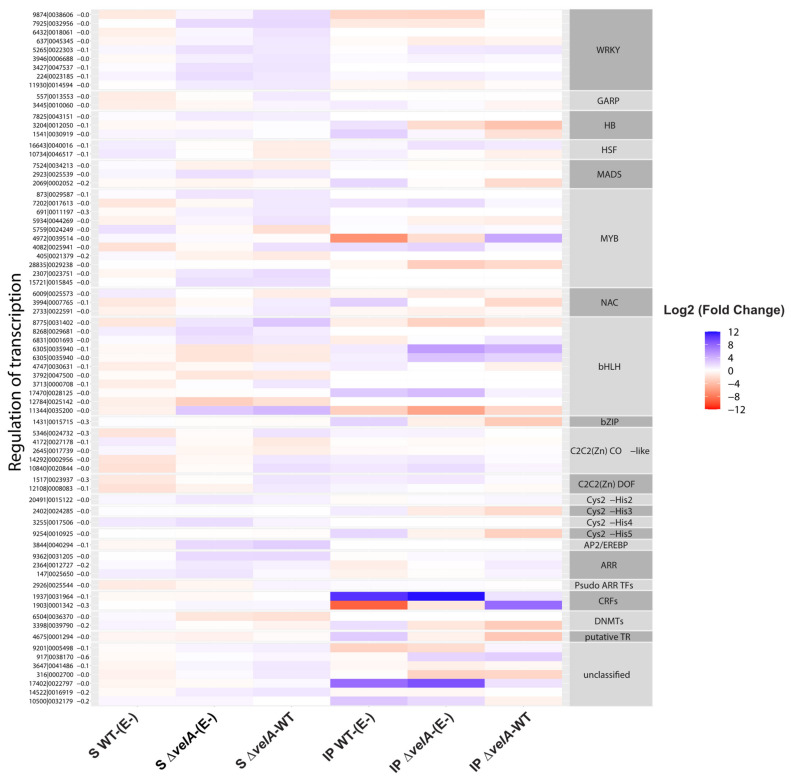
Distribution of predicted genes that encode different transcription factors. GARP: made of ARR−B and G2−like, HB: hemoglobin, HSF: heat shock factors, MADS: MADS−box transcriptional factors, MYB: myeloblastosis, NAC: NAM (no apical meristem, Petunia), ATAF1−2 (Arabidopsis thaliana activating factor), and CUC2 (cup−shaped cotyledon, Arabidopsis), bHLH: Basic Helix−Loop-Helix, bZIP: basic leucine zipper, CRFs: cytokinin response factors, DNMTs: DNA methylation.

Of genes predicted to be transcription factors, 76 genes were expressed differentially in one of the comparisons. Although each group of transcription factors have a different pattern of expression, most were up-regulated in seedling comparisons (S Δ*velA*-WT), whereas the opposite was seen for mature plant comparisons (IP Δ*velA*-WT) (Figure 5). 

Of 130 predicted genes that encode enzymes involved in nucleotide metabolism (synthesis, degradation, and salvage), only 6 genes were differentially expressed (Table S3) in seedling and mature plant comparisons, suggesting this process is not important in the plant response to *E. festucae*.

Of four DEGs predicted to be involved in starch synthesis, two of them were only differentially expressed in mature comparisons (Table 1). One of these genes is a homologue of granule-bound starch synthase 1, *waxy*, in *Hordeum vulgare* [25] and was 9.3 times up-regulated in IP Δ*velA*-WT comparisons (Table 1). This up-regulation of starch synthase genes correlates with a previously reported microscopy analysis, which showed higher numbers of starch granules in the Δ*velA* mutant infected mature plants (Figure 9 in [6]). Another DEG involved in starch synthesis was a homologue of beta-amylase 9 from *Brachypodium distachyon* that was up-regulated in both WT and Δ*velA* mutant infected mature plants. Two other genes involved in starch metabolism were only differentially expressed in seedling comparisons, with a homologue of beta-amylase 6 being down-regulated in S WT-(E-) and a homologue of glycogenin-like starch initiation protein 2 that was up-regulated in the S Δ*velA*-WT comparison (Table 1). 

Of sugar metabolism genes (Table S4), ten DEGs were directly related to sucrose biosynthesis. A homologue of sucrose phosphate synthase 1 from *Arabidopsis* [26], involved in sucrose precursor degradation, was 8.7 times down-regulated in IP WT-(E-) but was not expressed in seedlings, showing the importance of sucrose metabolism in mature plants. There were six invertase genes, involved in the breakdown of sucrose to glucose and fructose, which were differentially expressed in at least one of the comparisons. One of them, a homologue of fructan exohydrolase from *Phleum pratense,* that acts as a cell wall invertase, was 6.6 times up-regulated in IP WT-(E-) but not differentially expressed in seedlings. Another category of sugar-metabolism-related genes are sugar transporters, of which ten of them were differentially expressed in at least one of the comparisons. Of these 10 genes, 8 were only differentially expressed in seedlings, with the other 2 only being differentially expressed in mature plants. One of these two genes was up-regulated in IP Δ*velA*-(E-) 5.6 times and the other one was up-regulated 14 and 16.7 times in S Δ*velA*-(E-) and IP WT-(E-), respectively (Table S4). 

Of 21 genes identified for encoding enzymes involved in photosynthesis reactions in *L. perenne,* 12 were differentially expressed in at least one of the comparisons (Table S5). Of these genes, 7 of them were only differentially expressed in seedlings but at much lower levels (maximum 3.7 folds) compared with mature plants (maximum 27.3 folds). 

Plant cell walls, the next layer after the cuticle, are made of embedded cellulose microfibrils in a matrix of pectin, hemicellulose, and cell-wall-associated proteins [27]. Of the 38 genes identified as involved in cell wall cellulose synthesis, only 6 genes were differentially expressed in at least one of the seedling comparisons. Three of them are cellulose synthase-like proteins [28]. One was down-regulated 12.3-fold (IP WT-(E-)), one was down-regulated 12.1-fold (IP Δ*velA*-(E-)), and one was down-regulated 4.2-fold (S Δ*velA*-(E-) (Table S6). 

Of all predicted genes to have cell wall degradation function (47 genes), there are 4 that differentially expressed in one of the comparisons (Table S5). One of them is a homologue to a mannan endo-1,4-beta-mannosidase 1 gene which was 20.4-fold up-regulated and 14.2-fold down-regulated in IP Δ*velA*-WT and IP WT-(E-) comparisons, respectively, but not differentially expressed in seedling comparisons. This enzyme is involved in breaking down the mannon polysaccharides in the plant cell walls [29].

Of all genes associated with cell wall modification (32 genes), 6 were differentially expressed in at least one of the comparisons, including 4 genes predicted to encode expansins and 2 genes predicted to encode xyloglucan endotransglucosylases (Table S5). Expansins, by breaking bonds between matrix glucans and cellulose microfibers, are involving in loosening the plant cell wall [30]. Of the four DEGs with homology to expansins, three were not significantly differentially expressed in either mature or seedling comparisons but one was highly down-regulated (10.2-fold) in the mature IP Δ*velA*-(E) association (Table S6). Xyloglucan endotransglucosylases are involved in re-ligating and breaking down xyloglucan polymers in plant cell walls of growing tissue (Yokoyama and Nishitani, 2001). One of the two DEGs with this function identified in this study was down-regulated 5.8-fold in mutant Δ*velA* infected plant compared with wild type infected plant (Table S6). 

### 3.4. Mutant Endophytes Change Secondary Metabolism in Their Host Plants 

Of 107 expressed ryegrass genes (consolidated from 361 genes encoding redundant proteins) predicted to encode enzymes involved in secondary metabolism, 55 were differentially expressed in at least one of the comparisons (Table S7). Genes involved in lignin and terpenoids production were two of the secondary metabolites with the most significant differences. 

**Figure 6:**
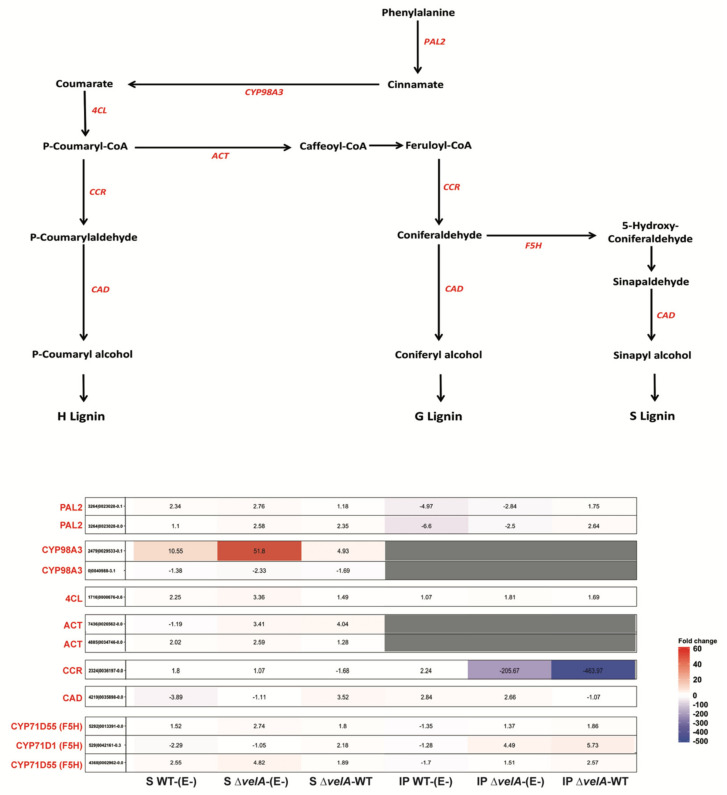
Expression changes of genes involved in the lignin biosynthetic pathway in ryegrass hosts in different comparisons. Schematic pathway showing fold changes of genes involved in lignin biosynthesis in different comparisons (based on MapMan). Sig., statistically significant (fold change ≥ 2 and FDR ≤ 0.05); PAL2, phenylalanine ammonia-lyase 2; CYP98A3, cytochrome P450, family 98; 4CL, 4−coumarate:CoA ligase; ACT1, agmatine coumaroyltransferase-1; ROMT−17, tricin synthase 2; CCR, cinnamoyl−CoA reductase; CAD, cinnamyl-alcohol dehydrogenase; F5H, ferulate 5−hydroxylase (including CYP71B, cytochrome P450, family 71, subfamily B; cytochrome P450 71D1; CYP71D55, premnaspirodiene oxygenase). http://www.plantphysiol.org/content/153/3/895, accessed on 1 April 2019.

Plants often deposit lignin at the infection site of a pathogen, reinforcing the cell wall as one of the most important defence mechanisms [31]. Interestingly, all 12 genes predicted to encode enzymes involved in lignin biosynthesis were differentially expressed, with the majority being up-regulated in seedling comparisons (Table S7 and Figure 6), including one gene (a homologue of CYP98A3) involved in catalysing cinnamate to courmarate in the lignin biosynthesis pathway [32] (Figure 6) being up-regulated 10.5 times in S WT-(E-) and 51.8 times in S Δ*velA*-(E-). In contrast, in mature plant comparisons, the majority of lignin biosynthesis genes were not differentially expressed (Figure 6); however, a homologue to cinnamoyl coA reductase (CCR) involved in lignin production was one of the most highly differentially expressed genes identified in this study, being down-regulated 205.6-fold in IP Δ*velA*-(E-) compared with only a 2.2-fold up-regulation in IP WT-(E-). This clearly demonstrates a significant difference in lignin production between wild type and mutant infected mature plants. 

Terpenoids are secondary metabolites with antifungal activities [33]. Of 22 genes associated with their biosynthesis, 6 genes were differentially expressed in at least one of the comparisons. These genes were mostly up-regulated in mutant infected seedlings and not expressed in wild type infected seedlings but were highly down-regulated in mature associations (Table S7). 

### 3.5. Infecting Ryegrass with ΔvelA E. Festucae Mutant Alters the Expression of Genes Responsible for Biotic and Abiotic Stresses 

Ryegrass transcriptomics showed that genes related to biotic and abiotic stress were influenced by *Epichloë* infection. 

Regarding abiotic stress-related genes, ryegrass infection with mutant Δ*velA E. festucae* strongly influenced temperature responsive genes, mostly in seedlings (Table S8). These included three cold stress peroxidase genes, one of which was highly down-regulated (516 folds) in seedlings inoculated with the Δ*velA* mutant (S Δ*velA*-(E-)) compared with only a 2.5-fold change in the wild type; this change was not seen in mature comparisons. Of the heat stress genes, seven genes were differentially expressed in at least one of the comparisons. Homologues of chaperone superfamily proteins were down-regulated in seedlings comparisons and up-regulated in mature comparisons (Table S8). RmlC-like cupins superfamily proteins (also called Germin) have superoxide dismutase (SOD) activity against extracellular superoxide radicals and act as defence proteins [34]. Of the five RmlC-like cupins identified from ryegrass, three were up-regulated in seedling comparisons but only one was differentially expressed (up-regulated) in mature comparisons (Table S8). 

Fifty-eight DEGs were identified that are predicted to be involved in response to biotic stress. These were chitinases, disease resistance proteins, pathogenesis-related proteins, and receptors (Table S9). Of 13 predicted chitinase genes in the ryegrass genome, 5 were differentially expressed in at least one of the comparisons. All were significantly up-regulated in the S Δ*velA*-(E-) comparison, but interestingly, only two were differentially expressed in mature plants (IP WT-(E-)) (Table S9). This demonstrates the importance of chitinases in both establishing infection by *Epichloë* and establishing a compatible interaction. Genes predicted to encode disease resistance proteins were classified into three groups based on their protein domain structure: coiled coil-nucleotide-binding site leucine-rich repeat (CC-NBS-LRR), nucleotide binding-adaptor shared by NOD-LRR proteins, APAF-1, R proteins, and CED4 (NB-ARC), and both LRR and NB-ARC. Most were highly differentially expressed in the mature comparisons (mostly down-regulated), but in the seedling comparisons, only a few were differentially expressed (slightly up-regulated). Interestingly, there was no overlap between seedlings and mature plants in DEG homologues to disease resistance proteins, which indicates possible development-stage dependency of the expression of each of these genes (Table S9). There are 14 gene homologues to pathogenesis-related (PR) proteins which were differentially expressed (up-regulated) in the S Δ*velA*-(E-) comparison but not expressed in mature plant comparisons (Table S9).

In response to invading microbes, plants produce different types of ROS that can play different roles in plant defence. One of the ROS functions is acting as an antimicrobial agent to protect the plant against invading microbes, and another is acting as one of the first signals to induce other plant responses against invading pathogens [35,36]. 

In this study, 33 differentially expressed genes in at least one of the comparisons were found that belong to three groups of enzymes involved in ROS production and detoxification, including peroxidases, glutathione S-transferases (GSTs), and other enzymes involved in redox state (Table S10). Interestingly, 31 of these genes were only differentially expressed in seedling comparisons. Of 100 predicted genes that encode peroxidases, 12 were differentially expressed in one of the seedling comparisons compared with only 1 in mature comparisons; these include the 3 peroxidases previously identified for the cold stress responses. Half of these DEGs are down-regulated in seedlings (range of 2 to 221 folds) infected with the Δ*velA* mutant compared with the wild type (Table S10). Because peroxidases are involved in the degradation of H_2_O_2_ molecules [37], a higher level of down-regulation of peroxidase genes in Δ*velA* mutant inoculated seedlings could result in increased H_2_O_2_ production, which is corroborated by our previous histology study that showed higher H_2_O_2_ production in seedlings inoculated with the Δ*velA* mutant [6,38]. A broad range of functions were shown for plant GSTs, including responses to biotic and abiotic stresses, transporters of anthocyanin, xenobiotics, and herbicide detoxification, auxin homeostasis, hydrogen peroxide detoxification, tyrosine metabolism, and regulation of apoptosis [39,40]. Of 57 genes detected for GSTs in ryegrass, 14 are differentially expressed in seedlings and 1 in mature comparisons (Table S10). Of these 14 genes, 10 were up-regulated in the infected seedlings with Δ*velA* compared with the wild type. This up-regulation of GST genes in seedlings was opposite to peroxidases which were significantly down-regulated. Lastly, a DEG highly up-regulated in seedlings infected with the mutant is predicted to encode a haemoglobin-like protein involved in scavenging nitric oxide [41] (Table S7). In total, the expression of different genes involved in ROS production possibly leads to increased ROS production in seedlings infected with *velA* mutants, whereas a decrease in ROS production would be predicted for mature plants. 

During plant responses to stress, plant hormones have an important regulatory role. Analysis of DEGs predicted to encode enzymes involved in hormone biosynthesis (abscisic acid, auxin, brassinosteroids, jasmonic acid, salicylic acid, gibberellins, and ethylene) showed that in the IP Δ*velA*-WT comparison, all hormone biosynthetic genes were either down-regulated or not differentially expressed (Table S11), but in the seedling comparisons, they were mostly up-regulated (Table S11). One of these hormones is brassinosteroid (BR) which increases plant resistance to biotic and abiotic stresses [42,43]. With higher concentrations of BRs, ROS production is increased, and this increases plant defence against pathogens. Conversely, lower concentrations of BRs promote plant growth by regulating other growth promoters [44,45]. There are only four DEGs involved in BR metabolism which are predicted to encode cytochrome P450 enzymes engaged in the biosynthesis of sterols, which are precursors for BR biosynthesis. These four genes were only differentially expressed in seedling comparisons and one of them has one of the highest fold changes in hormone metabolism genes and was down-regulated 107-fold (Table S11). 

During plant–microbe interactions, the balance between jasmonic acid (JA) and salicylic acid (SA) regulates plant responses against microbe invasion [46]. Of 37 genes predicated to be involved in JA biosynthesis in perennial ryegrass, 11 were differentially expressed (Table S11), including genes predicted to encode 6 isoforms of 13-lipoxygenase (LOX), 3 jasmonic acid carboxyl methyltransferase (JMT), and 2 of OPDA (12-Oxo-PDA) (Table S11). OPDA is involved in the biosynthesis of JA, LOX catalyses the first step in JA synthesis, and JMT methylates JA to the inactive methyl (+)-7-isojasmonate [47]. Interestingly, the 6 LOX genes differentially expressed in seedlings were only up-regulated in the S Δ*velA*-WT comparison and not differentially expressed in the S WT-(E-) comparison (Table S11). On the other hand, LOX genes differentially expressed in the mature plant comparisons were down-regulated in both the IP WT-(E-) and IP Δ*velA*-WT comparisons, but in IP Δ*velA*-WT to a much higher level. Regarding the three JMT genes, only one of them was differentially expressed in the IP Δ*velA*-(E-) (up-regulated 4.9 folds), one was differentially expressed in the S Δ*velA*-(E-) (up-regulated 2.3 folds), and the last one was only differentially expressed in the S Δ*velA*-WT comparison (down-regulated 2.3 folds). Of the two OPDA genes, one was differentially expressed in the IP WT-(E-) comparison (down-regulated 6.4 folds) but the other one was only differentially expressed in the S Δ*velA*-(E-) comparison (up-regulated 2.2 folds) (Table S11). 

Of 26 genes predicted to be involved in SA biosynthesis, only one was differentially expressed in the S Δ*velA*-WT comparison (up-regulated 2.4 folds) (Table S11). This gene is predicted to encode salicylic acid glucosyltransferase (UGT74F), which is engaged in both activation and deactivation of SA by transferring a glycosyl group [48]. 

## 4. Discussion

*Epichloë* fungi form bioprotective endophytic symbioses with many cool-season grasses, including agriculturally important forage grasses such as PRG. These endophytic associations have a very important influence on plant growth and interaction with environmental stresses [49,50,51,52,53]. In addition, certain studies have shown that *Epichloë* can reprogram host plant transcription [12,13,14,15,54,55,56].

Velvet (*velA*) is an important gene in filamentous fungi that influences several processes, such as fungal growth and metabolism and resistance to various stresses [57,58,59,60,61,62], and we have previously reported its importance in the symbiosis of *E. festucae* with PRG [6,9,38]. Deletion of *velA* in *E. festucae* changed a mutualistic interaction into an antagonistic/pathogenic one, providing a useful system to study pathways important in regulating the symbiosis between *E. festucae* and PRG [6,8,9]. In this paper, we identified these pathways by performing comparative transcriptomics using PRG inoculated with an antagonistic Δ*velA E. festucae* mutant compared with mutualistic symbiotic WT associations. In addition to performing transcriptomics on mature plants, we also, for the first-time, compared the PRG transcriptome of developing seedlings (two weeks old). Our results showed that PRG-transcriptome reprograming was dependent on both the growth stage and whether the interaction was antagonistic (Δ*velA*) or mutualistic (WT). Major pathways that changed, in particular, were those related to defence, such as lignin and ROS production, and those related to RNA processes, notably including WRKY transcription factors.

Overall, in this study, 1158 genes (6.45%) were identified as differentially expressed in at least one of the comparisons. Additionally, 400 genes were only differentially expressed in mature plants, 667 only in seedlings, and 91 genes were common to both seedlings and mature plants. Previous studies using transcriptomics to study grass–*Epichloë* interactions showed a broad range of DEGs, from as low as 2% to a high of 30% and were related to the tissue type, the stage of growth, and the methods of analysis [10,12,13,14,15,54,55], making interpretation of the results across studies difficult. 

The identification of DEGs in this study, using different fungal associations (E-, Δ*velA*, and WT) at two different stages of plant growth (seedling and mature plant), has shed additional light on how *Epichloë* influences its host PRG. There was greater than 2 times DEGs in S Δ*velA*-(E-) compared with S WT-(E-) but this difference was not detected in similar comparisons of mature plants (Figure 1A), indicating that *Epichloë* deficient in *velA* are severely compromised in establishing a compatible symbiosis during the early stages of infection. This is likely due to an increase in defence responses and associated genes during the early stages of infection which ultimately leads to significant (70%) seedling death [6]. Conversely, in mature plants, there is a much lower defence response, leading to reduced numbers of DEGs and survival of the plants. Studying common DEGs between different comparisons (Figure 1C) showed that PRG expressed a different set of genes against Δ*velA* and WT *E. festucae*, in addition to expressing a unique set of genes in each of the development stages. This could relate to the condition-dependent regulatory role of VelA in *E. festucae* whereby it was suggested that different protein complexes and/or different post-translational modifications/localizations may occur under different conditions [9]. Nevertheless, this is the first study in PRG showing growth-stage dependency of the transcriptome during interaction with *E. festucae,* and is similar to studies on the tall festucae–*E. coenophiala* interaction in which tissue-specific expression by both the fungus and the plant is shown [14,55].

Functional annotation studies of significant DEGs showed the involvement of 30 out of 51 different metabolic pathways which are associated with primary metabolism, secondary metabolism, and response to stresses. Most of the DEGs with primary metabolic functions were found to be involved in nucleotide metabolism, sugar metabolism-related mechanisms, and plant defence responses such as lignin and ROS production. Regarding nucleotide metabolism, it seems RNA metabolism has a much higher importance than DNA metabolism in the PRG–*E. festucae* interaction because more than 11 percent (77 of 650 genes) of the genes related to RNA metabolism were differentially expressed in at least one of the comparisons but only 4.6 percent (6 of 130 genes) of the genes related to DNA metabolism are differentially expressed. In this group of genes, there are 76 genes predicted to be transcription factors that belong to different groups, including WRKY transcriptions factors. WRKYs are known for their role in response to abiotic stresses, wounding, and pathogen infection in different plants [63]. Interestingly, these genes have totally different directions of expression in seedlings compared with mature plants, with most being up-regulated in S Δ*velA*-WT but down-regulated in IP Δ*velA*-WT. These different patterns show that different metabolic activities and functions are activated during the early stages of infection compared with later stages. The importance of WRKY transcription factors in the *Epichloë* interaction with grasses has also been shown for *E. coenophiala*–tall fescue [14], especially under water deficit [64] and in *E. festucae*–PRG [12].

In this study, genes related to different mechanisms of sugar metabolism, including photosynthesis, starch production, and sucrose biosynthesis, were differentially expressed. Genes related to starch biosynthesis showed possible higher production of starch granules in the infected plants, especially in Δ*velA* infected plants, which correlates to our previous microscopy analysis [6]. It is known that plants use starch as a stress response mechanism by remobilizing glucose from sorted starch which can provide energy and carbon during stress [65]. This suggests that surviving PRG plants infected with the Δ*velA* mutant may use starch production as a defence mechanism. Investigating the expression of the genes related to sugar metabolism showed that there is a possibly higher number of sugars such as sucrose produced in the surviving mature plants compared with the seedling stage. There was also a higher level of expression of genes related to photosynthesis in mature plants infected with the Δ*velA* mutant which leads to a concomitantly higher production of sugars. This is likely a response to the unlimited and abnormal fungal growth in the incompatible interaction leading to increased fungal biomass [6] and an increased requirement for carbon, since fungal transcriptomics indicates that the Δ*velA* mutant fungi are undergoing starvation [9].

Another important plant function influenced by fungal infection is cell wall metabolism. The plant cell wall is the first layer of fungal interaction and is thus important in defining the nature of the symbiosis between *Epichloë* and its host grass. Interestingly, enzymes that are involved in breaking down the cell wall were up-regulated in mature plants infected with the Δ*velA* mutant, suggesting this is a defence response under stress [66]. More degradation of the cell wall of the Δ*velA* mutant infected plants likely result in a thinner cell wall which has also been shown by Dupont et al. [12] using PRG infected with a different *E. festucae* mutant.

For genes related to secondary metabolism, around 50 percent were involved in lignin and terpenoid production, both of which are involved in plant defence responses against pathogens. Our results showed that lignin biosynthetic genes were not differentially expressed in mature plants but rather were up-regulated in seedlings, especially in the Δ*velA* mutant infected seedlings. However, in our previous study of lignin deposition using microscopy, we did not observe any obvious difference between inoculated seedlings with Δ*velA* and wild type [6,38]. Another important factor related to plant defence responses is ROS production. Overall, DEGs related to ROS were up-regulated in seedlings inoculated with the Δ*velA* mutant. This correlates with our previous study in which we showed higher levels of H_2_O_2_ production in the Δ*velA* mutant inoculated seedlings compared with the wild type. In contrast, in the mature plant comparisons, genes related to ROS production were generally not differentially expressed or were altered in a way that would be expected to lead to a decrease in ROS production. Other groups of plant defence and biotic stresses-related functions were also identified. These included 58 genes comprising chitinases, disease resistance proteins, pathogenesis-related proteins, and receptors that were almost entirely up-regulated in seedlings infected with the Δ*velA* mutant but were not differentially expressed or were down-regulated in mature plant associations. This suggests that in the early stages of the interaction, the Δ*velA* mutant is recognised as a pathogen, leading to a greater transcriptomic response and a higher death rate as we previously reported [6]. On the other hand, in the surviving mature plants, there appears to be a reduced plant response which leads to a stable but incompatible interaction compared with wild type infected plants [6]. Correlations with the plant-defence response transcription profiles hormonal pathways were also identified. These included genes related to brassinosteroid, jasmonic acid, and salicylic acid which, similar to defence responses, were up-regulated in seedlings infected with the Δ*velA* mutant but down-regulated or not differentially expressed in the mature plant comparisons. This also suggests there is a pathogenic interaction in the Δ*velA* mutant-associated seedlings.

Using a combination of different fungal strains (WT and Δ*velA* mutant) in different plant developmental stages, we uncovered the dynamic effects of endophyte infection on PRG gene expression. Endophyte infection, leading to either antagonistic or mutualistic interactions, has an important influence on the PRG transcriptome through activating/deactivating important pathways, especially stress responses. Dissecting these pathways in more detail will be a major focus in future research.

## Figures and Tables

**Table 1:** DEGs predicted to encode enzymes engaged in starch synthesis. Fold changes shown in bold are statistically significant (FDR ≤ 0.05); changed more than two times.

			Fold Change					
Gene ID	Bincode Name	Best Annotation	S WT-(E-)	S ΔvelA-(E-)	S Δ*velA*-WT	IP WT-(E-)	IP Δ*velA*-(E-)	IP Δ*velA*-WT
13063|0011328-0.1	‘major CHO metabolism.synthesis.starch.starch synthase’	starch synthase 1	−1.12	1.04	1.16	**−7.45**	1.25	**9.31**
1617|0046842-0.0	‘major CHO metabolism.degradation.starch.starch cleavage.beta amylase’	beta-amylase 9-like	−1.83	−1.89	−1.03	**7.20**	**5.13**	−1.40
1952|0042706-0.4	‘major CHO metabolism.degradation.starch.starch cleavage.beta amylase’	beta-amylase 6	**−2.24**	−1.55	1.45	−1.02	−1.25	−1.22
6792|0008466-0.0	‘cell wall.hemicellulose synthesis.glucuronoxylan’	plant glycogenin-like starch initiation protein 2	2.44	**4.00**	1.64	−1.34	−1.23	1.09

## Data Availability

HiSeq Illumina sequencing included 36 raw sequence datasets that have been deposited into the NCBI SRA database with the BioProject ID PRJNA578737.

## Supplementary Materials

The following supporting information can be downloaded at: https://www.mdpi.com/article/10.3390/jof9020190/s1, Table S1: General description of mRNA-sequencing results; Table S2: DEGs encode proteins involved in RNA metabolism (RNA transcription, regulation of transcription, RNA processing); Table S3: DEGs encode proteins involved in nucleotide metabolism (Synthesis, degradation and salvage); Table S4: DEGs predicted to encode enzymes engaged in sugar metabolism; Table S5: DEGs predicted to encode enzymes engaged in photosynthesis; Table S6: DEGs predicted to encode enzymes associated in plant and fungal cell wall; Table S7: DEGs encode proteins involved in secondary metabolites biosynthesis; Table S8: DEGs encode proteins involved in abiotic stresses; Table S9: DEGs encode proteins involved in biotic stresses; Table S10: DEGs encode proteins involved in ROS production and detoxification; Table S11: DEGs encode proteins involved in hormone metabolism.
