# Supplementary material for: Comparative Transcriptomics Profiling of Perennial Ryegrass Infected with Wild Type or a ΔvelA Epichloë festucae Mutant Reveals Host Processes Underlying Mutualistic versus Antagonistic Interactions
Source: J Fungi (Basel). 2023 Feb 1;9(2):190. doi: 10.3390/jof9020190 (PMC9959145; doi:10.3390/jof9020190)
Supplement: Supplementary file 1 [file jof-09-00190-s001.zip › jof-2090892-supplementary.pdf]

Table S1- General description of mRNA-sequencing results

**mRNA-sequencing reads**	**In planta**
Total read number	810,694,678
Number of total reads after quality trimming	809,938,396
Percentage of total reads after quality trimming	99.91%
Number of mapped reads	724,293,326
Proportion of mapped reads (% of trimmed reads)	89.43%
Number of reads mapped to Endophyte genome	13,360,773
Percentage of mapped fungal reads to total mapped reads	1.84%
Number of reads mapped to Ryegrass genome	710,932,553
Percentage of mapped ryegrass reads to total mapped reads	98.16%

Table S2- DEGs encode proteins involved in RNA metabolism (RNA transcription, regulation of transcription, RNA processing). Fold changes show in bold are statistically significant (FDR≤0.05) changed more than two times. Empty fold change cells are representative of not expressed gene in both compared strains.

Gene ID	Bincode Name	Fold Change					
		S WT-(E-)	S ΔvelA-(E-)	S Δ*velA*-WT	IP WT-(E-)	IP Δ*velA*-(E-)	IP Δ*velA*-WT
3844|0040294-0.1	'RNA.regulation of transcription.AP2/EREBP, APETALA2/Ethylene-responsive element binding protein family'	-1.44	**3.87**	**5.56**			
147|0025650-0.0	'RNA.regulation of transcription.ARR'	1.93	**2.47**	1.28	-1.71	-1.11	1.54
2364|0012727-0.2	'RNA.regulation of transcription.ARR'	1.27	**2.16**	1.70	-2.08	-1.08	1.92
9362|0031205-0.0	'RNA.regulation of transcription.ARR'	-1.03	**3.69**	**3.81**	-1.09	1.27	1.38
11344|0035200-0.0	'RNA.regulation of transcription.bHLH,Basic Helix-Loop-Helix family'	-1.79	**7.05**	**12.60**	**-7.96**	**-47.80**	**-6.00**
12784|0025142-0.0	'RNA.regulation of transcription.bHLH,Basic Helix-Loop-Helix family'	-2.01	**-7.71**	**-3.83**			
17470|0028125-0.0	'RNA.regulation of transcription.bHLH,Basic Helix-Loop-Helix family'	-1.17	-1.10	1.07	**6.65**	**11.29**	1.70
3713|0000708-0.1	'RNA.regulation of transcription.bHLH,Basic Helix-Loop-Helix family'	-2.10	1.11	**2.34**			
3792|0047500-0.0	'RNA.regulation of transcription.bHLH,Basic Helix-Loop-Helix family'	-1.21	**-2.87**	-2.38			
4747|0030631-0.1	'RNA.regulation of transcription.bHLH,Basic Helix-Loop-Helix family'	**-2.07**	-1.37	1.51	1.88	1.06	-1.76
6305|0035940-0.0	'RNA.regulation of transcription.bHLH,Basic Helix-Loop-Helix family'	-1.28	**-3.22**	**-2.52**	1.75	**8.44**	**4.82**
6305|0035940-0.1	'RNA.regulation of transcription.bHLH,Basic Helix-Loop-Helix family'	-1.37	**-3.23**	**-2.35**	2.08	**33.65**	**16.19**
6831|0001693-0.0	'RNA.regulation of transcription.bHLH,Basic Helix-Loop-Helix family'	1.24	**2.74**	**2.21**	-2.19	1.11	2.43
8268|0029681-0.0	'RNA.regulation of transcription.bHLH,Basic Helix-Loop-Helix family'	1.93	**3.65**	1.90			
8775|0031402-0.0	'RNA.regulation of transcription.bHLH,Basic Helix-Loop-Helix family'	**-2.85**	**2.52**	**7.18**	-2.18	**-6.70**	-3.07
1431|0015715-0.3	'RNA.regulation of transcription.bZIP transcription factor family'	1.11	-1.09	-1.20	**4.81**	-2.00	**-9.60**
10840|0020844-0.0	'RNA.regulation of transcription.C2C2(Zn) CO-like, Constans-like zinc finger family'	**-3.76**	-1.21	**3.12**	2.30	3.38	1.47
14292|0002956-0.0	'RNA.regulation of transcription.C2C2(Zn) CO-like, Constans-like zinc finger family'	**-3.22**	-1.36	**2.36**	1.84	2.15	1.17
2645|0017739-0.0	'RNA.regulation of transcription.C2C2(Zn) CO-like, Constans-like zinc finger family'	1.35	-1.51	**-2.04**	-1.22	-1.12	1.09
4172|0027178-0.1	'RNA.regulation of transcription.C2C2(Zn) CO-	1.99	-1.27	**-2.52**	-1.09	-1.31	-1.20

	like, Constans-like zinc finger family'						
5346|0024732-0.3	'RNA.regulation of transcription.C2C2(Zn) CO-like, Constans-like zinc finger family'	**-3.21**	-1.18	**2.73**	1.52	1.60	1.05
12108|0008083-0.1	'RNA.regulation of transcription.C2C2(Zn) DOF zinc finger family'	**-3.65**	-1.71	**2.13**	1.42	1.12	-1.27
1517|0023937-0.3	'RNA.regulation of transcription.C2C2(Zn) DOF zinc finger family'	**-2.77**	-1.09	**2.54**	1.87	2.31	1.23
20491|0015122-0.0	'RNA.regulation of transcription.C2H2 zinc finger family'	1.39	**2.36**	1.70	-1.19	1.15	1.37
2402|0024285-0.0	'RNA.regulation of transcription.C2H2 zinc finger family'	1.10	1.11	1.01	2.05	-2.37	**-4.86**
3255|0017506-0.0	'RNA.regulation of transcription.C2H2 zinc finger family'	2.21	**3.33**	1.50			
9254|0010925-0.0	'RNA.regulation of transcription.C2H2 zinc finger family'	1.08	-1.06	-1.15	4.31	-1.72	**-7.43**
1903|0001342-0.3	'RNA.regulation of transcription.Chromatin Remodeling Factors'	1.07	1.44	1.35	**-623.32**	-2.91	**209.35**
1937|0031964-0.1	'RNA.regulation of transcription.Chromatin Remodeling Factors'	-1.32	-1.35	-1.03	**1511.81**	**3326.33**	2.61
3398|0039790-0.2	'RNA.regulation of transcription.DNA methyltransferases'	1.54	-1.09	-1.68	3.17	-2.83	**-8.99**
6504|0036370-0.0	'RNA.regulation of transcription.DNA methyltransferases'	1.29	**-2.94**	**-3.78**			
3445|0010060-0.0	'RNA.regulation of transcription.G2-like transcription factor family, GARP'	**-2.12**	-1.44	1.47	1.90	1.20	-1.58
557|0013553-0.0	'RNA.regulation of transcription.G2-like transcription factor family, GARP'	**-2.27**	-1.08	**2.10**	-1.02	1.03	1.04
1541|0030919-0.0	'RNA.regulation of transcription.HB,Homeobox transcription factor family'	1.46	1.62	1.11	**5.11**	1.40	-3.65
3204|0012050-0.1	'RNA.regulation of transcription.HB,Homeobox transcription factor family'	-1.37	-1.23	1.12	2.76	-4.65	**-12.88**
7825|0043151-0.0	'RNA.regulation of transcription.HB,Homeobox transcription factor family'	1.16	**2.12**	1.83			
10734|0046517-0.1	'RNA.regulation of transcription.HSF,Heat-shock transcription factor family'	**2.23**	1.07	**-2.09**	1.73	-1.09	-1.89
16643|0040016-0.1	'RNA.regulation of transcription.HSF,Heat-shock transcription factor family'	1.94	-1.12	**-2.17**	1.33	2.80	2.12
2069|0002052-0.2	'RNA.regulation of transcription.MADS box transcription factor family'	-1.27	-1.57	-1.24	4.13	-1.15	**-4.74**
2923|0025539-0.0	'RNA.regulation of transcription.MADS box transcription factor family'	1.43	**3.08**	2.15			

7524|0034213-0.0	'RNA.regulation of transcription.MADS box transcription factor family'	1.21	-1.78	**-2.16**	1.18	-1.20	-1.42
2307|0023751-0.0	'RNA.regulation of transcription.MYB domain transcription factor family'	-1.50	**2.42**	**3.63**			
28835|0029238-0.0	'RNA.regulation of transcription.MYB domain transcription factor family'	1.07	1.03	-1.04	-1.55	**-8.26**	**-5.33**
405|0021379-0.2	'RNA.regulation of transcription.MYB domain transcription factor family'	1.31	-1.80	**-2.36**			
5759|0024249-0.0	'RNA.regulation of transcription.MYB domain transcription factor family'	**3.12**	-1.28	**-3.99**	1.13	1.36	1.21
5934|0044269-0.0	'RNA.regulation of transcription.MYB domain transcription factor family'	-1.80	1.48	**2.65**	1.17	-1.83	-2.13
691|0011197-0.3	'RNA.regulation of transcription.MYB domain transcription factor family'	-1.30	1.74	**2.25**			
15721|0015845-0.0	'RNA.regulation of transcription.MYB-related transcription factor family'	1.09	**3.22**	**2.96**			
4082|0025941-0.0	'RNA.regulation of transcription.MYB-related transcription factor family'	**-3.71**	-1.27	**2.92**	3.32	**4.59**	1.38
4972|0039514-0.0	'RNA.regulation of transcription.MYB-related transcription factor family'	1.34	1.17	-1.14	**-103.96**	-4.47	**23.24**
7202|0017613-0.0	'RNA.regulation of transcription.MYB-related transcription factor family'	**-2.87**	-1.32	**2.18**	2.56	3.54	1.38
873|0029587-0.1	'RNA.regulation of transcription.MYB-related transcription factor family'	1.13	**2.56**	**2.26**			
2733|0022591-0.0	'RNA.regulation of transcription.NAC domain transcription factor family'	-1.79	1.18	**2.10**	-1.43	-1.90	-1.34
3994|0007765-0.1	'RNA.regulation of transcription.NAC domain transcription factor family'	**-2.67**	-1.39	1.93	**5.28**	1.01	**-5.20**
6009|0025573-0.0	'RNA.regulation of transcription.NAC domain transcription factor family'	1.99	-1.04	**-2.08**	-1.54	-2.48	-1.61
2926|0025544-0.0	'RNA.regulation of transcription.Psudo ARR transcription factor family'	**-2.36**	-1.54	1.53	1.24	1.16	-1.07
4675|0001294-0.0	'RNA.regulation of transcription.putative transcription regulator'	-1.47	-1.74	-1.18	**6.04**	-1.78	**-10.73**
10500|0032179-0.2	'RNA.regulation of transcription.unclassified'	1.40	1.40	-1.00	**7.22**	3.85	-1.87
14522|0016919-0.2	'RNA.regulation of transcription.unclassified'	-1.29	1.89	**2.43**	-1.34	-1.39	-1.03
17402|0022797-0.0	'RNA.regulation of transcription.unclassified'	-1.57	-1.25	1.26	**195.64**	**512.61**	2.74
316|0002700-0.0	'RNA.regulation of transcription.unclassified'	-1.73	1.23	**2.13**	-1.11	**-6.47**	**-5.83**
3647|0041486-0.1	'RNA.regulation of transcription.unclassified'	-1.58	1.44	**2.28**	-1.39	-1.95	-1.40
917|0038170-0.6	'RNA.regulation of transcription.unclassified'	-1.26	1.12	1.41	-1.27	4.36	**5.56**
9201|0005498-0.1	'RNA.regulation of transcription.unclassified'	-1.19	1.52	1.81	**-6.51**	-4.40	1.48

11930|0014594-0.0	'RNA.regulation of transcription.WRKY domain transcription factor family'	-1.07	**2.05**	**2.21**	-1.55	-1.84	-1.19
224|0023185-0.1	'RNA.regulation of transcription.WRKY domain transcription factor family'	1.50	**3.34**	**2.22**	1.27	2.03	1.60
3427|0047537-0.1	'RNA.regulation of transcription.WRKY domain transcription factor family'	1.15	**2.86**	**2.50**			
3946|0006688-0.0	'RNA.regulation of transcription.WRKY domain transcription factor family'	-1.31	1.82	**2.38**	1.20	1.54	1.29
5265|0022303-0.1	'RNA.regulation of transcription.WRKY domain transcription factor family'	1.37	**2.95**	**2.15**	-1.07	2.22	2.38
637|0045345-0.0	'RNA.regulation of transcription.WRKY domain transcription factor family'	-1.46	1.53	**2.23**	-1.31	-2.10	-1.61
6432|0018061-0.0	'RNA.regulation of transcription.WRKY domain transcription factor family'	-1.96	1.36	**2.66**			
7925|0032956-0.0	'RNA.regulation of transcription.WRKY domain transcription factor family'	-1.04	**3.82**	**3.98**	-2.50	-2.71	-1.08
9874|0038606-0.0	'RNA.regulation of transcription.WRKY domain transcription factor family'	**-2.50**	1.48	**3.68**	**-6.12**	**-6.54**	-1.07
4193|0041308-0.0	'RNA.RNA binding'	1.22	1.26	1.03	2.54	**-569.81**	**-1581.76**
1189|0015287-0.3	'RNA.transcription'	-1.12	-1.05	1.06	**14.01**	**19.44**	1.39
15129|0001576-0.0	'RNA.processing.RNA helicase'	-1.18	1.25	1.47	**260.84**	**105.58**	-2.42

Table S3- DEGs encode proteins involved in nucleotide metabolism (Synthesis, degradation and salvage). Fold changes show in bold are statistically significant (FDR≤0.05) changed more than two times.

Gene ID	Bincode Name	Fold Change					
		**S WT-(E-)**	**S ΔvelA-(E-)**	**S Δ*velA*-WT**	**IP WT-(E-)**	**IP Δ*velA*-(E-)**	**IP Δ*velA*-WT**
18772|0007075-0.0	'nucleotide metabolism.salvage.phosphoribosyltransferases.aprt'	-1.65	1.30	**2.15**	1.18	1.18	-1.01
2374|0043192-0.1	'nucleotide metabolism.degradation.pyrimidine.beta-ureidopropionase'	1.31	1.12	-1.16	**-8.39**	-2.21	3.79
2441|0047435-0.0	'nucleotide metabolism.deoxynucleotide metabolism.pseudouridine synthase'	**-2.43**	-1.36	1.79	**5.55**	1.09	**-5.08**
4196|0041311-0.2	'nucleotide metabolism.salvage.phosphoribosyltransferases.aprt'	1.55	**2.46**	1.59	-1.58	-1.26	1.26
6732|0019325-0.0	'nucleotide metabolism.degradation.purine'	**-3.36**	-1.34	**2.50**	-1.04	-3.94	-3.79
8947|0012172-0.0	'nucleotide metabolism.synthesis.pyrimidine.orotate phosphoribosyltransferase'	1.41	1.86	1.32	-1.49	3.57	**5.33**
18772|0007075-0.0	'nucleotide metabolism.salvage.phosphoribosyltransferases.aprt'	-1.65	1.30	**2.15**	1.18	1.18	-1.01
2374|0043192-0.1	'nucleotide metabolism.degradation.pyrimidine.beta-ureidopropionase'	1.31	1.12	-1.16	**-8.39**	-2.21	3.79

Table S4- DEGs predicted to encode enzymes engaged in sugar metabolism. Fold changes show in bold are statistically significant (FDR≤0.05) changed more than two times. Empty fold change cells are representative of not expressed gene in both compared strains.

			Fold Change					
**Gene ID**	**Bincode Name**	**Best annotation**	**S WT-(E-)**	**S ΔvelA-(E-)**	**S Δ*velA*-WT**	**IP WT-(E-)**	**IP Δ*velA*-(E-)**	**IP Δ*velA*-WT**
8128|ref0015167-0.0	'major CHO metabolism synthesis sucrose (SPS)'	Sucrose phosphate synthase 1 [*Arabidopsis taliana*]				**-8.72**	-1.56	**5.57**
5904|ref0034294-0.1	'major CHO metabolism synthesis sucrose (SPS)'	Sucrose-6-phosphate phosphohydrolase [Hordeum vulgare subsp. vulgare]	1.17	**2.17**	1.85	-2.05	1.10	2.26
12649|ref0035145-0.0	'major CHO metabolism degradation sucrose hexokinase'	hexokinase-7 [Brachypodium distachyon]	-2.02	1.29	**2.62**	1.13	-1.37	-1.55
5327|ref0035434-0.0	'major CHO metabolism degradation sucrose invertases cell wall'	fructan exohydrolase [Phleum pratense]	-1.77	-1.03	1.72	**6.61**	-1.10	**-7.30**
731|ref0019652-0.3	'major CHO metabolism degradation sucrose invertases cell wall'	fructan exohydrolase [Phleum pratense]	-1.70	1.26	**2.14**			
406|ref0021378-0.3	'major CHO metabolism degradation sucrose invertases cell wall'	cell wall invertase [Lolium perenne]	-1.29	1.56	**2.01**	-2.38	1.15	2.73
9463|ref0034934-0.0	'major CHO metabolism degradation sucrose invertases cell wall'	cell wall invertase [Triticum aestivum]	1.27	**2.44**	1.93	-1.99	-1.03	1.94
1673|ref0036117-0.0	'major CHO metabolism degradation sucrose invertases vacuolar'	fructosyltransferase-like protein [Lolium perenne]	-1.95	-1.62	1.21	**-9.06**	**-17.12**	-1.89
671|ref0020303-0.0	'major CHO metabolism degradation sucrose invertases vacuolar'	putative fructosyltransferase1 [Lolium perenne]	1.74	-1.02	-1.76	-2.13	2.75	**5.88**
6955|ref0011933-0.0	'transport sugars sucrose'	Sucrose transport protein SUT5 [Triticum urartu]	**2.27**	-1.08	**-2.47**	1.04	-1.16	-1.21
13424|ref0032032-0.0	'transport.sugars'	PREDICTED: sugar transport protein 13 [Setaria italica]	1.04	1.10	1.06	1.97	**5.62**	2.85
245|ref0012158-1.0	'transport.sugars'	hexose transporter [Oryza sativa Japonica Group]	-1.16	-1.01	1.15	**16.68**	**14.04**	-1.18
13641|ref0014757-0.0	'transport.sugars'	Sugar carrier protein C [Triticum urartu]	-1.00	**2.04**	**2.05**	1.65	1.06	-1.55
18487|ref0015440-0.0	'transport.sugars'	sugar transport protein 5-like isoform X1 [Brachypodium distachyon]	**-2.30**	-1.03	**2.24**	1.40	4.58	3.27
5190|ref0021123-0.3	'transport.sugars'	predicted protein [Hordeum vulgare subsp. vulgare]	-2.02	1.54	**3.12**	-3.15	-2.02	1.56
6199|ref0042523-0.0	'transport.sugars'	predicted protein [Hordeum vulgare subsp. vulgare]	-2.02	1.26	**2.54**	-1.93	1.07	2.07
8121|ref0015158-0.0	'transport.sugars'	sugar transport protein 14 [Brachypodium distachyon]	-1.81	1.34	**2.42**	-1.60	-1.19	1.34
8522|ref0019595-0.1	'transport.sugars'	Sugar transport protein 13 [Aegilops tauschii]	-1.76	1.18	**2.08**	-2.26	-1.06	2.13
8701|ref0036885-0.0	'transport.sugars'	MFS transporter [Hordeum vulgare subsp. vulgare]	-1.36	1.84	**2.51**	-2.38	-1.55	1.54

Table S5- DEGs predicted to encode enzymes engaged in photosynthesis. Fold changes show in bold are statistically significant (FDR≤0.05) changed more than two times. Empty fold change cells are representative of not expressed gene in both compared strains.

Gene ID	Bincode Name	Best annotation	Fold Change					
			**S WT-(E-)**	**S ΔvelA-(E-)**	**S Δ*velA*-WT**	**IP WT-(E-)**	**IP Δ*velA*-(E-)**	**IP Δ*velA*-WT**
3163|0009846-0.1	'PS light reaction photosystem I LHC-I'	photosystem I light harvesting complex gene 2	-1.93	1.04	**2.01**	2.00	1.05	-1.90
7424|0044379-0.1	'PS light reaction photosystem I LHC-I'	photosystem I light harvesting complex gene 2	**-2.28**	-1.35	1.68	1.38	1.03	-1.34
1452|0003114-0.3	'PS light reaction photosystem II LHC-II'	Chlorophyll a-b binding protein 1B, chloroplastic [Aegilops tauschii]	**-3.74**	-1.22	**3.07**	2.43	1.59	-1.53
1452|0003114-0.5	'PS light reaction photosystem II LHC-II'	Chlorophyll a-b binding protein 1B, chloroplastic [Aegilops tauschii]	-2.55	1.18	**3.01**	**22.27**	**5.37**	-4.14
1998|0021188-0.0	'PS light reaction photosystem II LHC-II'	chlorophyll a-b binding protein, chloroplastic [Brachypodium distachyon]	**-2.16**	-1.20	1.80	1.52	1.25	-1.22
228|0023193-1.3	'PS light reaction photosystem II LHC-II'	chlorophyll a-b binding protein of LHCII type 1 [Brachypodium distachyon]	-1.98	1.12	**2.23**	2.27	1.74	-1.31
399|0000946-0.5	'PS light reaction photosystem II LHC-II'	Chlorophyll a-b binding protein CP26, chloroplastic [Aegilops tauschii]	**-2.22**	-1.21	1.83	1.64	1.25	-1.32
7637|0001662-0.1	'PS light reaction photosystem II PSII polypeptide subunits'	Photosystem II 1 kDa polypeptide, chloroplast [Salmo salar]	-1.70	-1.46	1.17	1.78	**-5.60**	**-9.95**
7637|0001662-0.2	'PS light reaction photosystem II PSII polypeptide subunits'	Photosystem II 1 kDa polypeptide, chloroplastic [Aegilops tauschii]	-1.92	-1.32	1.46	**5.46**	-4.65	**-25.44**
21159|0002244-0.0	'PS light reaction state transition'	unnamed protein product [Triticum aestivum]				-1.00	**-8.87**	**-8.83**
22186|0033836-0.0	'PS light reaction state transition'	putative vesicle associated membrane protein [Triticum aestivum]	**2.97**	2.29	-1.30	-2.87	-1.96	1.47
4713|0037794-0.0	'PS light reaction state transition'	myosin-15-like isoform X1 [Brachypodium distachyon]	1.21	1.05	-1.15	**-18.29**	**-27.73**	-1.52

Table S6- DEGs predicted to encode enzymes associated in plant and fungal cell wall. Fold changes show in bold are statistically significant (FDR≤0.05) changed more than two times. Empty fold change cells are representative of not expressed gene in both compared strains.

Gene ID	Bincode Name	Best annotation	Fold change					
			**S WT-(E-)**	**S ∆velA-(E-)**	**S ∆*velA*-WT**	**IP WT-(E-)**	**IP ∆*velA*-(E-)**	**IP ∆*velA*-WT**
10004|0012901-0.0	'cell wall.cellulose synthesis.cellulose synthase'	cellulose synthase-like D3 [Arabidopsis thaliana]				**-12.18**	-2.43	**5.05**
12401|0030411-0.0	'cell wall.cellulose synthesis.cellulose synthase'	cellulose synthase-like D3 [Arabidopsis thaliana]	1.25	1.46	1.15	-1.63	**-12.32**	**-7.67**
1704|0031283-0.0	'cell wall.cellulose synthesis.cellulose synthase'	cellulose synthase like G2 [Arabidopsis thaliana]	-1.48	**-4.18**	**-2.73**			
2515|0039698-0.1	'cell wall.cellulose synthesis.COBRA'	predicted protein [Hordeum vulgare subsp. vulgare]	1.59	**3.46**	**2.05**	-1.83	1.10	2.04
246|0012161-0.1	'cell wall.cellulose synthesis'	hypothetical protein F775_1266 [Aegilops tauschii]	**-2.54**	**-4.38**	-1.74			
6792|0008466-0.0	'cell wall.hemicellulose synthesis.glucuronoxylan'	hypothetical protein F775_1266 [Aegilops tauschii]	2.44	**4.00**	1.81	-1.34	-1.23	1.03
2462|0011551-0.0	'cell wall.degradation.mannan-xylose-arabinose-fucose'	predicted protein [Hordeum vulgare subsp. vulgare]	**2.62**	**2.49**	-1			
5328|0035447-0.0	'cell wall.degradation.mannan-xylose-arabinose-fucose'	xyloglucan xyloglucosyl transferase [Hordeum vulgare]	-1.64	-1.07	1.54	**5.24**	**11.63**	2.23
5359|0042439-0.0	'cell wall.degradation.mannan-xylose-arabinose-fucose'	mannan endo-1,4-beta-mannosidase 1 [Brachypodium distachyon]	1.53	-1.29	-1.97	**-14.22**	1.42	**20.39**
634|0045342-0.5	'cell wall.degradation.pectate lyases and polygalacturonases'	hypothetical protein ZEAMMB73_269375 [Zea mays]	2.01	**2.46**	1.2			
16353|0013246-0.0	'cell wall.modification'	expansin B4 [Arabidopsis thaliana]	1.85	-1.18	**-2.17**	-2.22	**-10.25**	**-4.98**
10034|0030944-0.0	'cell wall.modification'	expansin A15 [Arabidopsis thaliana]	-1.07	**-2.65**	**-2.48**			
5014|0044908-0.1	'cell wall.modification'	expansin A1 [Arabidopsis thaliana]	1.48	**2.19**	1.47			
1899|0018801-0.0	'cell wall.modification'	expansin-A21-like [Brachypodium distachyon]	**2.11**	1.15	-1.84			
1776|0013307-0.1	'cell wall.modification'	xyloglucan endotransglucosylase [Arabidopsis thaliana]	1.62	1.13	-1.43	1.92	-3.11	**-5.84**
2451|0017008-0.0	'cell wall.modification'	xyloglucan endotransglucosylase [Hordeum vulgare]	-1.35	1.82	**2.47**	2.24	-2.22	**-4.93**
4019|0041942-0.5	'cell wall.pectin*esterases.acetyl esterase'	Pectinacetylesterase family protein [Arabidopsis thaliana]	**-2.39**	1.34	**3.19**	1.24	1.74	1.42

Table S7- DEGs encode proteins involved in secondary metabolites biosynthesis. Fold changes show in bold are statistically significant (FDR≤0.05) changed more than two times. Empty fold change cells are representative of not expressed gene in both compared strains.

Gene ID	Bincode Name	Best annotation	Fold Change					
			S WT-(E-)	S ΔvelA -(E-)	S Δ*velA* -WT	IP WT-(E-)	IP Δ*velA* -(E-)	IP Δ*velA* -WT
1716|0000676-0.6	secondary metabolism.phenylpropanoids.lignin biosynthesis.4CL'	OPC-8:0 CoA ligase1	**2.25**	**3.36**	1.49	1.07	1.81	1.69
0|0040988-3.1	'secondary metabolism.phenylpropanoids.lignin biosynthesis.C4H'	cytochrome P450, family 98, subfamily A, polypeptide 3	-1.38	**-2.33**	-1.69			
2479|0029533-0.1	'secondary metabolism.phenylpropanoids.lignin biosynthesis.C4H'	cytochrome P450, family 98, subfamily A, polypeptide 3	**10.55**	**51.80**	**4.93**			
2324|0036197-0.0	'secondary metabolism.phenylpropanoids.lignin biosynthesis.CCR1'	cinnamoyl coa reductase	1.80	1.07	-1.68	2.24	**-205.67**	**-463.97**
4219|0035898-0.0	secondary metabolism.phenylpropanoids.lignin biosynthesis.CCR1'	NAD(P)-binding Rossmann-fold superfamily protein	**-3.89**	-1.11	**3.52**	2.84	2.66	-1.07
4368|0002962-0.0	'secondary metabolism.phenylpropanoids.lignin biosynthesis.F5H'	cytochrome P450, family 71, subfamily B, polypeptide 34	**2.55**	**4.82**	1.89	-1.70	1.51	2.57
529|0042161-0.3	secondary metabolism.phenylpropanoids.lignin biosynthesis.F5H'	Cytochrome P450 superfamily protein	**-2.29**	-1.05	**2.18**	-1.28	4.49	**5.73**
5292|0013391-0.0	'secondary metabolism.phenylpropanoids.lignin biosynthesis.F5H'	cytochrome P450, family 71, subfamily B, polypeptide 34	1.52	**2.74**	1.80	-1.35	1.37	1.86
4885|0034746-0.0	secondary metabolism.phenylpropanoids.lignin biosynthesis.HCT'	HXXXD-type acyl-transferase family protein	2.02	**2.59**	1.28			
7436|0026562-0.0	'secondary metabolism.phenylpropanoids.lignin biosynthesis.HCT'	HXXXD-type acyl-transferase family protein	-1.19	**3.41**	**4.04**			

3264|0023028-0.0	'secondary metabolism.phenylpropanoids.lignin biosynthesis.PAL'	phenylalanine ammonia-lyase 2	1.10	**2.58**	**2.35**	**-6.60**	-2.50	2.64
3264|0023028-0.1	'secondary metabolism.phenylpropanoids.lignin biosynthesis.PAL'	phenylalanine ammonia-lyase 2	**2.34**	**2.76**	1.18	**-4.97**	-2.84	1.75
1105|0016983-0.7	'secondary metabolism.isoprenoids.terpenoids'	Terpenoid cyclases/Protein prenyltransferases superfamily protein	1.02	**-2.62**	**-2.66**			
15392|0002671-0.0	'secondary metabolism.isoprenoids.terpenoids'	terpene synthase-like sequence-1,8-cineole				**13.51**	**7.34**	-1.84
2163|0015757-0.0	'secondary metabolism.isoprenoids.terpenoids'	Terpenoid cyclases/Protein prenyltransferases superfamily protein	1.64	**2.14**	1.30			
3397|0039783-0.5	'secondary metabolism.isoprenoids.terpenoids'	terpene synthase-like sequence-1,8-cineole				-2.94	**-5.83**	-1.98
408|0021371-0.2	'secondary metabolism.isoprenoids.terpenoids'	Terpenoid cyclases/Protein prenyltransferases superfamily protein	-1.83	1.51	**2.76**	**-5.37**	-2.06	2.61
529|0042161-0.1	'secondary metabolism.isoprenoids.terpenoids'	terpene synthase-like sequence-1,8-cineole	-1.84	**15.05**	**27.66**	-1.31	**-88.26**	**-67.71**
15239|0044829-0.0	secondary metabolism.flavonoids.anthocyanins.anthocyanidin reductase'	dihydroflavonol 4-reductase	**2.34**	**2.52**	1.08	-1.93	-1.74	1.11
2994|0021678-0.1	'secondary metabolism.flavonoids.anthocyanins.anthocyanidin reductase'	dihydroflavonol 4-reductase	-1.72	1.03	1.77	1.46	**-7.80**	**-11.39**
5717|0000643-0.0	'secondary metabolism.flavonoids.anthocyanins'	2-oxoglutarate (2OG) and Fe(II)-dependent oxygenase superfamily protein	-1.13	**3.58**	**4.06**	-2.55	**-5.23**	-2.05
16995|0020958-0.0	'secondary metabolism.flavonoids.chalcones.naringenin-chalcone synthase'	Chalcone and stilbene synthase family protein	1.17	-1.51	-1.77	**-4.84**	2.13	**10.29**
2884|0002567-0.0	'secondary metabolism.flavonoids.chalcones'	Chalcone and stilbene synthase family protein	1.85	**38.23**	**20.71**			
8997|0019391-0.0	'secondary metabolism.flavonoids.dihydroflavonols.dihydroflavonol 4-reductase'	NAD(P)-binding Rossmann-fold superfamily protein	-1.68	**-4.28**	**-2.55**			
10510|0001618-0.0	'secondary metabolism.flavonoids.dihydroflavonols.flavonoid 3''-monooxygenase'	cytochrome P450, family 71, subfamily A, polypeptide 15	**-2.55**	1.02	**2.61**	1.15	-1.36	-1.56
11445|0015996-0.0	secondary metabolism.flavonoids.dihydroflavonols.flavonoid 3''-monooxygenase'	cytochrome P450, family 71 subfamily B, polypeptide 7	-1.66	**-2.12**	-1.28	-1.12	1.53	1.72

11649|0038902-0.1	'secondary metabolism.flavonoids.dihydroflavonols.flavonoid 3''-monooxygenase'	cytochrome P450, family 706, subfamily A, polypeptide 6	**-2.23**	-1.38	1.62	1.59	1.50	-1.06
15453|0008137-0.1	'secondary metabolism.flavonoids.dihydroflavonols.flavonoid 3''-monooxygenase'	cytochrome P450, family 71, subfamily B, polypeptide 35	1.18	**-2.37**	**-2.80**	**7.02**	**17.80**	2.54
4211|0043078-0.0	'secondary metabolism.flavonoids.dihydroflavonols.flavonoid 3''-monooxygenase'	cytochrome P450, family 706, subfamily A, polypeptide 6	1.27	**-2.26**	**-2.86**			
4396|0019374-0.0	'secondary metabolism.flavonoids.dihydroflavonols.flavonoid 3''-monooxygenase'	cytochrome P450, family 706, subfamily A, polypeptide 6	1.53	**5.65**	**3.68**	-3.73	-1.80	2.07
4481|0040498-0.1	'secondary metabolism.flavonoids.dihydroflavonols.flavonoid 3''-monooxygenase'	cytochrome P450, family 706, subfamily A, polypeptide 6	1.15	**6.19**	**5.37**	**-5.74**	-2.10	2.73
4481|0040498-0.3	'secondary metabolism.flavonoids.dihydroflavonols.flavonoid 3''-monooxygenase'	cytochrome P450, family 706, subfamily A, polypeptide 6	1.46	**5.27**	**3.62**	-3.45	-1.20	2.89
6356|0041246-0.0	'secondary metabolism.flavonoids.dihydroflavonols.flavonoid 3''-monooxygenase'	cytochrome P450, family 71, subfamily B, polypeptide 35	-1.34	-1.91	-1.42	3.84	**12.61**	3.28
6412|0039669-0.0	'secondary metabolism.flavonoids.dihydroflavonols.flavonoid 3''-monooxygenase'	cytochrome P450, family 712, subfamily A, polypeptide 1	1.56	-1.63	**-2.54**	-2.36	-1.40	1.69
6509|0006070-0.0	'secondary metabolism.flavonoids.dihydroflavonols.flavonoid 3''-monooxygenase'	cytochrome P450, family 706, subfamily A, polypeptide 6	**-2.13**	-1.95	1.09	1.29	2.07	1.60
8705|0036889-0.0	'secondary metabolism.flavonoids.dihydroflavonols.flavonoid 3''-monooxygenase'	cytochrome P450, family 71, subfamily B, polypeptide 37	1.10	-1.25	-1.38	**-13.21**	**-4.81**	2.74
185|0000330-1.0	'secondary metabolism.flavonoids.dihydroflavonols'	2-oxoglutarate (2OG) and Fe(II)-dependent oxygenase superfamily protein	1.89	**6.05**	**3.19**	-3.51	1.34	**4.69**
185|0000330-1.1	'secondary metabolism.flavonoids.dihydroflavonols'	2-oxoglutarate (2OG) and Fe(II)-dependent oxygenase superfamily protein	-1.92	1.33	**2.55**	**-5.54**	**-11.61**	-2.10
2542|0011927-0.0	'secondary metabolism.flavonoids.dihydroflavonols'	2-oxoglutarate (2OG) and Fe(II)-dependent oxygenase superfamily protein	-1.18	**2.53**	**3.00**	-1.23	-1.35	-1.09
18564|0016146-0.0	'secondary metabolism.flavonoids.flavonols'	2-oxoglutarate (2OG) and Fe(II)-dependent oxygenase superfamily protein	1.06	1.24	1.16	-4.21	1.20	**5.07**
4527|0038439-0.0	'secondary metabolism.flavonoids.flavonols'	2-oxoglutarate (2OG) and Fe(II)-dependent oxygenase superfamily protein	1.89	**2.80**	1.48	-1.98	1.01	2.01

14366|0045001-0.0	'secondary metabolism.flavonoids.isoflavones.isoflavone reductase'	NmrA-like negative transcriptional regulator family protein	-1.98	1.04	**2.06**			
12006|0041597-0.0	'secondary metabolism.isoprenoids.carotenoids.carotenoid beta ring hydroxylase'	beta-carotene hydroxylase 2	1.19	1.28	1.07	1.07	**5.75**	**5.40**
6708|0024836-0.0	'secondary metabolism.isoprenoids.non-mevalonate pathway.DXS'	1-deoxy-D-xylulose 5-phosphate synthase 1	**2.40**	**4.49**	1.87	-2.22	1.03	2.29
snap_4308|0038962-0.15	'secondary metabolism.N misc.alkaloid-like'	FAD-binding Berberine family protein	1.33	1.57	1.18	**-5.29**	1.49	**7.88**
snap_4308|0038962-0.8	'secondary metabolism.N misc.alkaloid-like'	FAD-binding Berberine family protein	1.59	**3.13**	1.97			
133|0020071-1.2	'secondary metabolism.phenylpropanoids'	O-methyltransferase family protein	1.24	**2.28**	1.83			
160|0012850-0.0	'secondary metabolism.phenylpropanoids'	HXXXD-type acyl-transferase family protein	1.25	-1.93	**-2.41**			
1770|0013301-0.0	'secondary metabolism.simple phenols'	laccase 12	**-2.61**	**-6.04**	**-2.31**			
1770|0013301-0.1	'secondary metabolism.simple phenols'	laccase 12	-1.65	**-2.29**	-1.39			
5878|0040205-0.0	'secondary metabolism.sulfur-containing.glucosinolates.degradation.myrosinase'	beta glucosidase 15	1.50	-1.46	**-2.20**			
8235|0022638-0.0	'secondary metabolism.sulfur-containing.glucosinolates.synthesis.shared.CYP83B1 phenylacetaldoxime monooxygenase'	cytochrome P450, family 71, subfamily B, polypeptide 20	1.99	**2.99**	1.51	-1.94	-1.08	1.80
4851|0029305-0.4	'secondary metabolism.wax'	beta-ketoacyl reductase 2	1.81	**2.94**	1.62			
6296|0018632-0.0	'secondary metabolism.wax'	Fatty acid hydroxylase superfamily	-1.57	1.54	**2.43**	-1.16	2.16	2.50
7855|0036148-0.1	'secondary metabolism.wax'	beta-ketoacyl reductase 1	1.14	**-3.01**	**-3.43**			

Table S8- DEGs encode proteins involved in abiotic stresses. Fold changes show in bold are statistically significant (FDR≤0.05) changed more than two times. Empty fold change cells are representative of not expressed gene in both compared strains.

Gene ID	Bincode Name	Best annotation	Fold Change					
			**S WT-(E-)**	**S Δ*velA*-(E-)**	**S Δ*velA*-WT**	**IP WT-(E-)**	**IP Δ*velA*-(E-)**	**IP Δ*velA*-WT**
16888|0036561-0.0	'stress.abiotic.cold'	Peroxidase superfamily protein	-1.02	1.17	1.20	1.70	-5.08	-8.62
27094|0033264-0.0	'stress.abiotic.cold'	Peroxidase superfamily protein	-2.45	-516.21	-221.92			
3783|0029599-0.2	'stress.abiotic.cold'	Peroxidase superfamily protein	1.09	-5.11	-5.58			
7468|0021099-0.0	'stress.abiotic.drought/salt'	ERD (early-responsive to dehydration stress) family protein	1.35	1.21	-1.11	-2.29	12.34	28.26
11003|0019732-0.0	'stress.abiotic.heat'	HSP20-like chaperones superfamily protein	2.05	-1.07	-2.20			
15044|0029114-0.0	'stress.abiotic.heat'	Chaperone DnaJ-domain superfamily protein	2.33	-1.04	-2.43	-1.46	-1.85	-1.27
1856|0042057-0.0	'stress.abiotic.heat'	DNAJ heat shock N-terminal domain-containing protein	1.33	-1.03	-1.36	1.59	6.10	3.85
4444|0017402-0.2	'stress.abiotic.heat'	DNAJ heat shock N-terminal domain-containing protein	-2.62	-1.41	1.86	2.08	2.55	1.23
5674|0034274-0.1	'stress.abiotic.heat'	heat shock transcription factor A2	-1.57	-2.30	-1.46	4.32	6.18	1.43
6225|0038301-0.0	'stress.abiotic.heat'	Chaperone DnaJ-domain superfamily protein	-3.34	-2.31	1.45	3.88	-3.80	-14.74
679|0020295-0.0	'stress.abiotic.heat'	winged-helix DNA-binding transcription factor family protein	1.03	2.16	2.09	-1.71	-1.06	1.61
1235|0015297-0.2	'stress.abiotic.touch/wounding'	Wound-responsive family protein	-1.71	2.10	3.34	1.20	-1.63	-1.96
10578|0037683-0.0	'stress.abiotic.unspecified'	RmlC-like cupins superfamily protein	2.25	4.04	1.80	-1.16	-2.13	-1.84
13475|0026757-0.1	'stress.abiotic.unspecified'	unknown protein	-16.04	1.07	17.06			
1655|0023677-0.0	'stress.abiotic.unspecified'	RmlC-like cupins superfamily protein	2.27	2.37	1.05			
2106|0029322-0.1	'stress.abiotic.unspecified'	RmlC-like cupins superfamily protein	1.11	-1.11	-1.23	10.38	6.16	-1.69

557|0013553-0.2	'stress.abiotic.unspecified'	RmlC-like cupins superfamily protein	1.46	2.40	1.64			
557|0013553-0.5	'stress.abiotic.unspecified'	RmlC-like cupins superfamily protein	1.54	2.73	1.77			
6807|0019838-0.4	'stress.abiotic.unspecified'	RmlC-like cupins superfamily protein	-1.21	1.86	2.26			
10085|0011254-0.0	'stress.abiotic'	SPX domain gene 1	1.23	-1.67	-2.06	-1.39	1.07	1.49

Table S9- DEGs encode proteins involved in biotic stresses. Fold changes show in bold are statistically significant (FDR≤0.05) changed more than two times. Empty fold change cells are representative of not expressed gene in both compared strains.

Gene ID	Bincode Name	Best annotation	Fold Change					
			**S WT-(E-)**	**S ΔvelA-(E-)**	**S Δ*velA*-WT**	**IP WT-(E-)**	**IP Δ*velA*-(E-)**	**IP Δ*velA*-WT**
13893|0007097-0.0	'not assigned.unknown'	Pathogenesis-related protein Bet v	1.07	1.20	1.12	-2.89	**-8.95**	-3.10
snap_1310|0005233-0.18	'stress.biotic'	basic pathogenesis-related protein 1	-1.04	**2.29**	**2.38**			
snap_367|0009954-0.1	'stress.biotic'	CAP (Cysteine-rich secretory proteins, Antigen 5, and Pathogenesis-related 1 protein) superfamily protein	1.51	**3.14**	2.08			
snap_18280|0002333-0.0	'stress.biotic'	basic pathogenesis-related protein 1	**2.44**	**5.02**	**2.06**	2.53	1.03	-2.47
snap_27036|0021856-0.0	'stress.biotic'	basic pathogenesis-related protein 1	1.39	**2.21**	1.58	1.57	-1.15	-1.81
snap_10431|0035258-0.6	'stress.biotic'	CAP (Cysteine-rich secretory proteins, Antigen 5, and Pathogenesis-related 1 protein) superfamily protein	1.73	**2.92**	1.69	-2.03	-1.57	1.29
537|0024475-0.2	'not assigned.unknown'	Pathogenesis-related protein Bet v	1.57	**2.19**	1.39	-1.57	1.11	1.74
2380|0021883-0.0	'not assigned.unknown'	START-like domain Bet v	1.89	**2.39**	1.27			
8639|0033693-0.0	'not assigned.unknown'	START-like domain Bet v	1.68	**2.32**	1.38			
8639|0033693-0.1	'not assigned.unknown'	Pathogenesis-related protein Bet v	1.48	**2.16**	1.46	-2.07	-1.96	1.06
snap_17168|0014271-0.1	'stress.abiotic'	Pathogenesis-related thaumatin superfamily protein	**2.30**	**2.23**	-1.03	-1.13	1.01	1.14
snap_19370|0001080-0.0	'stress.abiotic'	Pathogenesis-related thaumatin superfamily protein	1.30	**2.48**	1.92	-1.31	-2.52	-1.92
snap_376|0040308-0.21	'stress.abiotic'	Pathogenesis-related thaumatin superfamily protein	2.01	**3.38**	1.68	**-7.13**	-2.22	3.21
509|0006343-0.3	'stress.biotic.PR-proteins'	receptor like protein 12				**5.86**	**7.09**	1.21
1247|0044125-0.3	'stress.biotic.respiratory burst'	NADPH/respiratory burst oxidase protein D	-1.23	**2.31**	**2.84**			
783|0026874-0.6	'stress.biotic.signalling.MLO-like'	Seven transmembrane MLO family protein	2.11	**5.35**	**2.53**	-2.29	-1.44	1.59
10465|0029861-0.0	'stress.biotic'	HXXXD-type acyl-transferase family protein	1.75	**7.07**	**4.05**	**-7.62**	-2.75	2.77

12352|0024047-0.0	'stress.biotic'	HXXXD-type acyl-transferase family protein	-1.29	1.05	1.35	3.56	**9.25**	2.60
17121|0039368-0.0	'stress.biotic'	Plant basic secretory protein (BSP) family protein	**2.39**	**4.43**	1.86	-1.61	1.55	2.50
11164|0016212-0.0	'stress.biotic'	Chitinase family protein	1.98	**3.15**	1.59	**-7.11**	-1.77	4.03
13325|0046918-0.0	'stress.biotic'	Chitinase family protein	2.32	**7.06**	**3.04**	**-4.63**	-4.33	1.07
1983|0003140-0.1	'stress.biotic'	Chitinase family protein	**4.55**	**11.80**	**2.60**	-3.25	1.12	3.65
21371|0046601-0.0	'stress.biotic'	Chitinase family protein	-1.19	**2.00**	**2.36**	-1.92	-1.12	1.72
25915|0013866-0.0	'stress.biotic'	Chitinase family protein	1.66	**3.14**	1.89	-2.28	-2.32	-1.02
5518|0030549-0.0	'stress.biotic'	LRR and NB-ARC domains-containing disease resistance protein	1.16	-1.16	-1.35	3.01	**9.09**	3.02
274|0017642-1.2	'stress.biotic'	LRR and NB-ARC domains-containing disease resistance protein	-1.11	1.08	1.20	**9.79**	4.11	-2.38
476|0026821-0.2	'stress.biotic'	LRR and NB-ARC domains-containing disease resistance protein	-1.35	1.50	**2.02**	-1.15	-1.00	1.15
10643|0016359-0.0	'stress.biotic'	NB-ARC domain-containing disease resistance protein	1.16	1.06	-1.09	1.43	**-4.98**	**-7.13**
11308|0010204-0.1	'stress.biotic'	Disease resistance protein (CC-NBS-LRR class) family	1.33	**4.66**	**3.50**			
31175|0012032-0.0	'stress.biotic'	NB-ARC domain-containing disease resistance protein	1.34	-1.67	**-2.23**	2.62	1.89	-1.38
3297|0040849-0.0	'stress.biotic'	RPS5-like 1	-1.18	-1.23	-1.05	**-25.74**	1.97	**50.73**
1595|0025675-0.2	'stress.biotic'	LRR and NB-ARC domains-containing disease resistance protein	-1.28	1.73	**2.21**	-1.30	2.81	3.64
4245|0027234-0.0	'stress.biotic'	Disease resistance protein (CC-NBS-LRR class) family				1.68	**-6.90**	**-11.59**
4364|0002974-0.2	'stress.biotic'	Disease resistance protein (CC-NBS-LRR class) family	1.40	**2.46**	1.76			
545|0031512-0.1	'stress.biotic'	LRR and NB-ARC domains-containing disease resistance protein	**-2.32**	-1.19	1.95	1.08	2.37	2.19
5593|0028879-0.1	'stress.biotic'	Disease resistance protein (CC-NBS-LRR class) family	-1.63	-1.50	1.09	**-192.8**	1.39	**266.3**
6532|0046890-0.2	'stress.biotic'	Disease resistance protein (CC-NBS-LRR class) family				-2.93	**-1623.0**	**-606.3**

695|0011193-0.3	'stress.biotic'	NB-ARC domain-containing disease resistance protein	1.21	1.05	-1.15	-1.30	**5.75**	**7.46**
7128|0030119-0.0	'stress.biotic'	Disease resistance protein (CC-NBS-LRR class) family	-1.10	-1.26	-1.14	**-7.19**	1.13	**8.12**
7492|0015655-0.0	'stress.biotic'	NB-ARC domain-containing disease resistance protein	1.63	**2.20**	1.35			
888|0020833-0.4	'stress.biotic'	basic chitinase	**2.61**	**4.82**	1.85	-2.07	-1.84	1.13
101|0001927-0.2	'not assigned.unknown'	Putative disease resistance protein RGA1	-2.04	1.22	**2.49**	2.11	-1.15	-2.44
10403|0017285-0.2	'not assigned.unknown'	Disease resistance protein RGA2	-1.38	1.34	1.84	**-31.41**	-2.86	**10.95**
14642|0039966-0.0	'not assigned.unknown'	unknown protein	-1.04	**-2.16**	**-2.08**			
19048|0042926-0.0	'not assigned.no ontology'	Leucine-rich repeat (LRR) family protein	-1.27	1.81	**2.30**	-2.18	-2.76	-1.27
6|0040996-1.2	'not assigned.unknown'	unknown protein	1.01	-1.40	-1.41	**-6.07**	-1.54	3.95
903|0007606-0.3	'stress.biotic'	NB-ARC domain-containing disease resistance protein	-2.18	1.16	**2.53**	-2.71	-2.05	1.32
12002|0041593-0.0	'stress'	cysteine-rich RLK (RECEPTOR-like protein kinase) 8	-2.03	1.21	**2.47**	-1.02	-2.48	-2.42
13207|0000774-0.0	'stress'	cysteine-rich RLK (RECEPTOR-like protein kinase) 10	-1.70	1.43	**2.43**	-1.28	-1.07	1.20
13207|0000774-0.1	'stress'	cysteine-rich RLK (RECEPTOR-like protein kinase) 10	-1.93	1.96	**3.79**	-2.28	-1.41	1.62
14476|0031662-0.0	'stress'	cysteine-rich RLK (RECEPTOR-like protein kinase) 4	-1.27	1.57	2.00	**6.03**	2.00	-3.01
15194|0021387-0.0	'stress'	cysteine-rich RLK (RECEPTOR-like protein kinase) 8	1.25	**2.48**	1.98	-1.58	-2.39	-1.52
1737|0036758-0.0	'stress'	S-locus lectin protein kinase family protein	-1.01	1.24	1.26	-1.10	**-99.74**	**-91.58**
2057|0007590-0.1	'stress'	cysteine-rich RLK (RECEPTOR-like protein kinase) 8	1.10	1.39	1.26	**-145.9**	-1.69	**86.31**
2891|0020639-0.1	'stress'	cysteine-rich RLK (RECEPTOR-like protein kinase) 8	**-2.11**	1.19	**2.50**	-1.02	-1.73	-1.69
3862|0004424-0.0	'stress'	cysteine-rich RLK (RECEPTOR-like protein kinase) 8	1.30	**2.41**	1.85	-2.02	-1.18	1.71
4226|0017958-0.2	'stress'	cysteine-rich RLK (RECEPTOR-like protein kinase) 4	-1.06	**2.58**	**2.74**	-1.31	-1.16	1.13
6395|0016207-0.0	'stress'	cysteine-rich RLK (RECEPTOR-like protein kinase) 19	1.47	1.74	1.18	-3.78	2.55	**9.64**

Table S10- DEGs encode proteins involved in ROS production and detoxification. Fold changes show in bold are statistically significant (FDR≤0.05) changed more than two times. Empty fold change cells are representative of not expressed gene in both compared strains.

Gene ID	Bincode Name	Best annotation	Fold Change					
			S WT-(E-)	S ΔvelA-(E-)	S Δ*velA*-WT	IP WT-(E-)	IP Δ*velA*-(E-)	IP Δ*velA*-WT
11920|0032529-0.0	'misc.peroxidases'	Peroxidase superfamily protein	-1.42	1.43	**2.02**			
13363|0021942-0.0	'misc.peroxidases'	Peroxidase superfamily protein	1.50	-1.48	**-2.21**			
191|0030881-1.2	'misc.peroxidases'	Peroxidase superfamily protein	1.22	**2.30**	1.89	-1.36	1.32	1.80
29|0012215-0.0	'misc.peroxidases'	Peroxidase family protein	-1.35	1.57	**2.12**	1.06	1.01	-1.05
13400|0019582-0.0	'misc.peroxidases'	Peroxidase superfamily protein	1.34	**2.15**	1.61			
3020|0043251-0.1	'misc.peroxidases'	Peroxidase superfamily protein	-1.06	**2.50**	**2.66**			
21563|0025807-0.0	'misc.peroxidases'	Peroxidase superfamily protein	-1.34	**-2.65**	-1.97			
2254|0004379-0.3	'misc.peroxidases'	Peroxidase superfamily protein	-1.71	**-7.17**	**-4.20**			
8229|0004594-0.0	'misc.peroxidases'	Peroxidase superfamily protein	1.48	**2.66**	1.80			
3783|0029599-0.2	'stress.abiotic.cold'	Peroxidase superfamily protein	1.09	**-5.11**	**-5.58**			
27094|0033264-0.0	'stress.abiotic.cold'	Peroxidase superfamily protein	**-2.45**	**-516.21**	**-221.92**			
16888|0036561-0.0	'stress.abiotic.cold'	Peroxidase superfamily protein	-1.02	1.17	1.20	1.70	**-5.08**	**-8.62**
8786|0035014-0.2	'redox.ascorbate and glutathione.glutathione'	glutathione peroxidase 7	**-3.73**	-1.18	**3.16**	3.75	4.30	1.15
30|0030220-2.0	'misc.glutathione S transferases'	glutathione S-transferase tau 7	**-2.26**	1.20	**2.71**	-2.31	-3.35	-1.45
1335|0041028-0.3	'misc.glutathione S transferases'	glutathione S-transferase tau 4	1.28	**3.09**	**2.42**	-2.67	1.06	2.82
4704|0007237-0.2	'misc.glutathione S transferases'	glutathione S-transferase 6	-1.87	1.11	**2.07**	1.68	1.91	1.14
4704|0007237-0.3	'misc.glutathione S transferases'	glutathione S-transferase 6	-1.99	1.11	**2.21**	1.27	-1.08	-1.37
17715|0008450-0.0	'misc.glutathione S transferases'	Glutathione S-transferase family protein	**-2.67**	**-2.84**	-1.07			
1565|0043379-0.0	'misc.glutathione S transferases'	Glutathione S-transferase family protein	1.35	-1.57	**-2.13**	-1.13	-2.94	-2.61
4382|0037315-0.0	'misc.glutathione S transferases'	glutathione S-transferase 6	**2.57**	**7.37**	**2.87**	-3.17	1.23	3.89
5539|0043047-0.0	'misc.glutathione S transferases'	Peroxidase superfamily protein	**2.48**	**21.58**	**8.70**	-3.01	-2.11	1.43
5539|0043047-0.1	'misc.glutathione S transferases'	Peroxidase superfamily protein	1.07	**4.52**	**4.21**	-3.94	-2.99	1.32

3511|0019559-0.2	'misc.glutathione S transferases'	glutathione S-transferase TAU 18	-1.23	**-2.49**	-2.03			
11527|0037060-0.0	'misc.glutathione S transferases'	glutathione S-transferase tau 4	-1.19	**2.36**	**2.80**	-2.30	-1.10	2.09
2254|0004379-0.5	'misc.glutathione S transferases'	glutathione S-transferase TAU 18	-1.53	1.01	1.55	**-8.07**	-4.22	1.91
869|0011605-0.1	'misc.glutathione S transferases'	Peroxidase superfamily protein	1.76	**2.68**	1.53			
28602|0004008-0.0	'misc.glutathione S transferases'	Glutathione S-transferase family protein	1.44	-1.61	**-2.31**	-1.12	1.00	1.12
8786|0035014-0.2	'redox.ascorbate and glutathione.glutathione'	glutathione peroxidase 7	**-3.73**	-1.18	**3.16**	3.75	4.30	1.15
5890|0031114-0.6	redox.thioredoxin'	tetratricopetide-repeat thioredoxin-like 2	1.16	1.31	1.13	1.70	**-3.72**	**-6.34**
7843|0005643-0.1	'redox.thioredoxin'	thioredoxin F-type 1	**-2.24**	-1.52	1.48	1.69	2.13	1.27
20777|0001419-0.0	'redox.ascorbate and glutathione'	cytochrome B5 isoform B	2.67	**5.55**	2.08			
3042|0007389-0.0	'redox.ascorbate and glutathione.ascorbate'	Plant L-ascorbate oxidase	1.09	**2.71**	**2.48**	1.62	-1.20	-1.94
542|0031513-0.2	'redox.heme'	haemoglobin 2	1.44	**15.83**	**11.07**			

Table S11- DEGs encode proteins involved in hormone metabolism. Fold changes show in bold are statistically significant (FDR≤0.05) changed more than two times. Empty fold change cells are representative of not expressed gene in both compared strains.

Gene ID	Bincode Name	Best annotation	Fold Change					
			S WT-(E-)	S ∆velA-(E-)	S ∆*velA*-WT	IP WT-(E-)	IP ∆*velA*-(E-)	IP ∆*velA*-WT
**Brassinosteroid (BR)**								
12035|0036245-0.0	'hormone metabolism.brassinosteroid.synthesis-degradation.sterols.CYP51'	cytochrome P450, family 707, subfamily A, polypeptide 4	**2.98**	**4.20**	1.41			
529|0042161-0.4	'hormone metabolism.brassinosteroid.synthesis-degradation.sterols.CYP51'	cytochrome P450, family 707, subfamily A, polypeptide 4	**-2.65**	**-4.45**	-1.68	1.18	-1.24	-1.46
6857|0012593-0.0	'hormone metabolism.brassinosteroid.synthesis-degradation.sterols.CYP51'	cytochrome P450, family 707, subfamily A, polypeptide 4	**2.54**	**2.21**	-1.15	-1.60	1.18	1.88
11890|0002733-0.0	'hormone metabolism.brassinosteroid.synthesis-degradation.sterols.CYP51'	cytochrome P450, family 707, subfamily A, polypeptide 4	-1.37	**-107.83**	**-79.88**			
**Jasmonic acid (JA)**								
3901|0030125-0.0	hormone metabolism.jasmonate.synthesis-degradation.12-Oxo-PDA-reductase'	12-oxophytodienoate reductase 1	-1.12	1.58	1.77	**-6.39**	-1.59	4.02
15130|0032139-0.0	'hormone metabolism.jasmonate.synthesis-degradation.12-Oxo-PDA-reductase'	12-oxophytodienoate reductase 2	1.33	**2.17**	1.63	-1.34	-2.56	-1.91
9293|0011514-0.0	'hormone metabolism.jasmonate.synthesis-degradation.jasmonate-O-methyltransferase'	S-adenosyl-L-methionine-dependent methyltransferases superfamily protein	1.45	-1.55	**-2.25**	1.55	1.03	-1.50
3223|0047938-0.2	'hormone metabolism.jasmonate.synthesis-degradation.jasmonate-O-methyltransferase'	S-adenosyl-L-methionine-dependent methyltransferases superfamily protein	-1.97	-1.38	1.42	1.46	**4.94**	3.38
1134|0047379-0.0	'hormone metabolism.jasmonate.synthesis-degradation.jasmonate-O-methyltransferase'	S-adenosyl-L-methionine-dependent methyltransferases superfamily protein	1.29	**2.27**	1.76	1.42	-1.49	-2.11

3877|0034879-0.1	'hormone metabolism.jasmonate.synthesis-degradation.lipoxygenase'	PLAT/LH2 domain-containing lipoxygenase family protein	-1.39	1.49	**2.07**	-2.92	-2.49	1.18
786|0026877-0.0	'hormone metabolism.jasmonate.synthesis-degradation.lipoxygenase'	PLAT/LH2 domain-containing lipoxygenase family protein	-1.30	**8.75**	**11.41**	**-6.92**	**-210.46**	**-30.41**
14669|0004090-0.0	'hormone metabolism.jasmonate.synthesis-degradation.lipoxygenase'	PLAT/LH2 domain-containing lipoxygenase family protein	1.05	**2.01**	1.92	-1.68	-1.88	-1.13
12318|0000453-0.2	'hormone metabolism.jasmonate.synthesis-degradation.lipoxygenase'	PLAT/LH2 domain-containing lipoxygenase family protein	-1.88	-1.30	1.45	**-15.85**	**-24.25**	-1.53
7715|0034244-0.1	'hormone metabolism.jasmonate.synthesis-degradation.lipoxygenase'	PLAT/LH2 domain-containing lipoxygenase family protein	1.21	1.66	1.37	**-5.02**	**-29.79**	**-5.93**
10098|0041679-0.1	'hormone metabolism.jasmonate.synthesis-degradation.lipoxygenase'	PLAT/LH2 domain-containing lipoxygenase family protein	-1.88	1.37	**2.58**	-1.16	-1.48	-1.28
**Salicylic acid (SA)**								
4452|0035376-0.1	'hormone metabolism.salicylic acid.synthesis-degradation'	UDP-glycosyltransferase 74 F1	-1.43	1.69	**2.42**	-1.41	1.42	2.01
**Abscisic acid (ABA)**								
812|0040415-0.2	'hormone metabolism.abscisic acid.signal transduction'	highly ABA-induced PP2C gene 2	-1.15	1.21	1.39	1.60	**6.27**	3.92
5258|0040140-0.0	'hormone metabolism.abscisic acid.synthesis-degradation'	aldehyde oxidase 2	1.08	-1.06	-1.15	**-9.09**	-2.31	3.94
7065|0030538-0.3	'hormone metabolism.abscisic acid.synthesis-degradation'	aldehyde oxidase 2	-1.14	1.20	1.37	**5.10**	2.08	-2.45
767|0012406-0.0	'hormone metabolism.abscisic acid.synthesis-degradation.degradation.8-hydroxylase'	cytochrome P450, family 707, subfamily A, polypeptide 3	-1.61	1.41	**2.27**	**5.78**	-1.17	**-6.76**
3566|0012299-0.0	'hormone metabolism.abscisic acid.synthesis-degradation.synthesis.short chain alcohol dehydrogenmase (ABA2)'	NAD	1.68	**3.45**	2.05			
658|0032726-0.3	'hormone metabolism.abscisic acid.synthesis-degradation.synthesis.zeaxanthin epoxidase'	FAD/NAD	**-3.50**	-1.26	**2.77**	2.74	3.02	1.10
**Auxin**								
2098|0032594-0.0	'hormone metabolism.auxin'	methyl esterase 18	-1.91	1.18	**2.25**	-1.10	1.87	2.06

765|0012408-0.2	'hormone metabolism.auxin.induced-regulated-responsive-activated'	Auxin-responsive GH3 family protein				-1.91	**-5.16**	-2.70
1613|0046846-0.1	'hormone metabolism.auxin.induced-regulated-responsive-activated'	NAD	1.39	-1.56	**-2.16**	-1.69	-1.76	-1.04
1566|0043380-0.0	'hormone metabolism.auxin.induced-regulated-responsive-activated'	NAD	-1.94	-1.58	1.23	**5.71**	2.66	-2.15
3065|0019997-0.0	'hormone metabolism.auxin.induced-regulated-responsive-activated'	NAD	**-3.46**	1.12	**3.88**	-1.14	-1.25	-1.09
3870|0034872-0.1	'hormone metabolism.auxin.induced-regulated-responsive-activated'	AUX/IAA transcriptional regulator family protein	-1.11	2.07	**2.31**			
7380|0034159-0.0	'hormone metabolism.auxin.induced-regulated-responsive-activated'	Auxin-responsive GH3 family protein	-1.16	**2.82**	**3.28**	-1.39	1.60	2.22
446|0044598-0.2	'hormone metabolism.auxin.induced-regulated-responsive-activated'	NAD	1.48	-1.58	**-2.34**			
16122|0031619-0.0	'hormone metabolism.auxin.induced-regulated-responsive-activated'	Auxin-responsive family protein	-1.43	1.41	**2.02**	-1.42	-2.14	-1.51
549|0031519-0.5	'hormone metabolism.auxin.signal transduction'	Auxin efflux carrier family protein	1.45	-1.50	**-2.17**	1.22	-1.43	-1.74
549|0031519-0.6	'hormone metabolism.auxin.signal transduction'	Auxin efflux carrier family protein	1.40	-1.61	**-2.27**	1.23	-2.02	-2.48
**Cytokinin (CK)**								
4523|0038435-snap-gene-0.14	'hormone metabolism.cytokinin.synthesis-degradation'	cytokinin oxidase/dehydrogenase 6	-1.98	1.56	**3.09**			
237|0040978-1.0	'hormone metabolism.cytokinin.synthesis-degradation'	UDP-glucosyl transferase 85A2	2.04	**7.70**	**3.78**	-2.37	1.43	3.39
12802|0010595-0.1	'hormone metabolism.cytokinin.synthesis-degradation'	cytokinin oxidase/dehydrogenase 6	1.76	**3.50**	1.99	1.25	1.20	-1.05
1477|0040490-0.2	'hormone metabolism.cytokinin.synthesis-degradation'	cytokinin oxidase/dehydrogenase 1	-1.56	1.64	**2.56**	-2.12	-2.04	1.04
21174|0004587-0.0	'hormone metabolism.cytokinin.synthesis-degradation'	UDP-glucosyl transferase 85A3	**-2.16**	1.01	**2.17**	4.27	-1.60	**-6.83**
569|0019014-0.3	'misc.cytochrome P450'	cytochrome P450, family 735, subfamily A, polypeptide 2	1.18	1.17	-1.01	**13.45**	**-127.43**	**-1871.42**
**Ethylene**								
19592|0025930-0.0	'hormone metabolism.ethylene.induced-regulated-responsive-activated'	Adenine nucleotide alpha hydrolases-like superfamily protein	**-2.72**	**2.95**	**8.03**			
730|0019653-0.3	'hormone metabolism.ethylene.signal transduction'	Integrase-type DNA-binding superfamily protein	-1.14	**2.65**	**3.03**			

14174|0020942-0.0	'hormone metabolism.ethylene.synthesis-degradation.1-aminocyclopropane-1-carboxylate oxidase'	2-oxoglutarate	2.16	**13.19**	**6.10**			
21938|0036377-0.0	'hormone metabolism.ethylene.synthesis-degradation.1-aminocyclopropane-1-carboxylate synthase'	ACC synthase 1	1.30	**2.46**	1.90			
**Gibberellic acid (GA)**								
2519|0039696-0.0	'hormone metabolism.gibberelin.synthesis-degradation.ent-kaurene oxidase'	Cytochrome P450 superfamily protein	1.40	**2.19**	1.57	1.03	1.12	1.09
4368|0002962-0.1	'hormone metabolism.gibberelin.synthesis-degradation.ent-kaurene synthase'	terpene synthase 04	2.06	**3.89**	1.89	-3.37	1.19	4.01
7191|0025967-0.1	'hormone metabolism.gibberelin.synthesis-degradation.GA2 oxidase'	gibberellin 2-oxidase	-1.64	1.36	**2.22**			
5374|0030002-0.0	'hormone metabolism.gibberelin.synthesis-degradation.GA2 oxidase'	gibberellin 2-oxidase 3	1.97	1.12	-1.76	3.69	-2.73	**-10.06**
3140|0022390-0.2	'hormone metabolism.gibberelin.synthesis-degradation.GA20 oxidase'	gibberellin 20 oxidase 2	-1.60	1.78	**2.84**			
**Not defined**								
2664|0005249-0.1	'hormone metabolism'	acetone-cyanohydrin lyase	**2.42**	**4.44**	1.84	**-23.36**	-2.42	**9.65**
2664|0005249-0.2	'hormone metabolism'	acetone-cyanohydrin lyase	**3.79**	**4.51**	1.19			
